# SMARCA5 interacts with NUP98-NSD1 oncofusion protein and sustains hematopoietic cells transformation

**DOI:** 10.1186/s13046-022-02248-x

**Published:** 2022-01-24

**Authors:** Zivojin Jevtic, Vittoria Matafora, Francesca Casagrande, Fabio Santoro, Saverio Minucci, Massimilliano Garre’, Milad Rasouli, Olaf Heidenreich, Giovanna Musco, Jürg Schwaller, Angela Bachi

**Affiliations:** 1 Functional Proteomics group, IFOM-FIRC Institute of Molecular Oncology, Milan, Italy; 2 Imaging Technological Development Unit, IFOM-FIRC Institute of Molecular Oncology, Milan, Italy; 3 Chromatin Alterations in Tumorigenesis Unit, European Institute of Oncology, Milan, Italy; 4 Department of Biosciences, University of Milan, Milan, Italy; 5 Prinses Maxima Center for Pediatric Oncology, Utrecht, The Netherlands; 6 Biomolecular NMR, IRCCS Ospedale San Raffaele, Milan, Italy; 7 Department for Biomedicine, University Children Hospital, Basel, Switzerland

**Keywords:** NUP98-NSD1, SMARCA5, Acute myeloid leukemia, Phase-separation, Interactomics

## Abstract

**Background:**

Acute myeloid leukemia (AML) is characterized by accumulation of aberrantly differentiated hematopoietic myeloid progenitor cells. The karyotyping-silent NUP98-NSD1 fusion is a molecular hallmark of pediatric AML and is associated with the activating FLT3-ITD mutation in > 70% of the cases. NUP98-NSD1 fusion protein promotes myeloid progenitor self-renewal in mice via unknown molecular mechanism requiring both the NUP98 and the NSD1 moieties.

**Methods:**

We used affinity purification coupled to label-free mass spectrometry (AP-MS) to examine the effect of NUP98-NSD1 structural domain deletions on nuclear interactome binding. We determined their functional relevance in NUP98-NSD1 immortalized primary murine hematopoietic stem and progenitor cells (HSPC) by inducible knockdown, pharmacological targeting, methylcellulose assay, RT-qPCR analysis and/or proximity ligation assays (PLA). Fluorescence recovery after photobleaching and b-isoxazole assay were performed to examine the phase transition capacity of NUP98-NSD1 in vitro and in vivo.

**Results:**

We show that NUP98-NSD1 core interactome binding is largely dependent on the NUP98 phenylalanine-glycine (FG) repeat domains which mediate formation of liquid-like phase-separated NUP98-NSD1 nuclear condensates. We identified condensate constituents including imitation switch (ISWI) family member SMARCA5 and BPTF (bromodomain PHD finger transcription factor), both members of the nucleosome remodeling factor complex (NURF). We validated the interaction with SMARCA5 in NUP98-NSD1^+^ patient cells and demonstrated its functional role in NUP98-NSD1/FLT3-ITD immortalized primary murine hematopoietic cells by genetic and pharmacological targeting. Notably, SMARCA5 inhibition did not affect NUP98-NSD1 condensates suggesting that functional activity rather than condensate formation per se is crucial to maintain the transformed phenotype.

**Conclusions:**

NUP98-NSD1 interacts and colocalizes on the genome with SMARCA5 which is an essential mediator of the NUP98-NSD1 transformation in hematopoietic cells. Formation of NUP98-NSD1 phase-separated nuclear condensates is not sufficient for the maintenance of transformed phenotype, which suggests that selective targeting of condensate constituents might represent a new therapeutic strategy for NUP98-NSD1 driven AML.

**Supplementary Information:**

The online version contains supplementary material available at 10.1186/s13046-022-02248-x.

## Background

Acute myeloid leukemia (AML) is characterized by accumulation of aberrantly differentiated hematopoietic myeloid progenitor cells [1]. Chromosomal translocations involving the *Nucleoporin 98* gene (*NUP98*) result in > 30 distinct gene fusions found in different hematological malignancies including AML [2, 3]. Functional studies have shown that NUP98 fusion proteins promote myeloid progenitor self-renewal and prevent their differentiation through mechanisms that are heavily influenced by the fusion partner [2, 3]. Moreover, several recent studies suggested that transformation of myeloid progenitors directly depends on phase transition capacity of NUP98 fusion proteins, mediated by the disordered N-terminal NUP98 moiety which is shared among all fusions [4–6].

The karyotyping-silent NUP98-NSD1 fusion is a molecular hallmark of pediatric AML present with high white blood cell counts, and is generally associated with additional mutations of which the activating FLT3-ITD mutation is found in > 70% of the cases [7, 8]. The NUP98-NSD1 fusion carries the N-terminus of NUP98, including its disordered phenylalanine-glycine (FG) repeats and GLEBS domains, and the C-terminus of NSD1 bearing five plant homeodomains (PHD) and one PHD finger-like Cys-His-rich domain (C5HCH or PHD6), one proline-tryptophan-proline domain (PWWP), and the lysine methyltransferase SET domain [9]. Retroviral NUP98-NSD1 promotes myeloid progenitor self-renewal in mice by maintaining the expression of *HoxA7*, *HoxA9*, *HoxA10, HoxB4, HoxB5, and Meis1* genes via a mechanism requiring both the NUP98 and the NSD1 moieties [10, 11]. It has been shown that the NUP98 moiety interacts with transcriptional coactivators EP300 and KMT2A (also known as MLL1) [10, 11]. PHD5 and C5HCH domains from the NSD1 moiety mediate binding of genomic loci, while their transcriptionally active state is ensured by SET domain-catalyzed H3K36me2 [10]. Despite these insights, the molecular mechanism of NUP98-NSD1 driven leukemogenesis remains poorly characterized.

Herein, we determined the NUP98-NSD1 nuclear interactome, and examined its dependency on specific domains of the fusion protein. We found that core interactome binding was largely dependent on the FG repeat domains which mediate formation of liquid-like phase-separated NUP98-NSD1 nuclear condensates. We identified condensate constituents including imitation switch (ISWI) family member SMARCA5 and BPTF (bromodomain PHD finger transcription factor), both members of the nucleosome remodeling factor complex (NURF), and validated the interaction with SMARCA5 remodeler in NUP98-NSD1^+^ patient cells. Furthermore, we demonstrated an important role of SMARCA5 in self-renewal of NUP98-NSD1 immortalized murine hematopoietic cells and regulation of *HoxA9* and *Meis1* proto-oncogene expression. As SMARCA5 knockdown/pharmacologic targeting did not affect formation of the NUP98-NSD1 nuclear condensates, we propose that a fully functional interactome is necessary to maintain the transformed state.

## Materials & methods

### Cell lines and lysate preparations

Cell lines were acquired from the American Type Culture Collection (ATCC) and cell culture media were obtained from Lonza. All media were supplemented with 10% fetal bovine serum (FBS). HEK-293 cells were grown in DMEM, while 32Dcl3 cells were grown in RPMI containing 10 ng/ml of murine IL-3. The cells were grown at 37 °C with 5% of CO_2_ and maintained according to manufacturer instructions. For transfection experiments**,** HEK293 cells were plated at 70% confluency. We utilized the calcium-phosphate method [12] with 10 μg of corresponding plasmids. The medium was changed after 8 h and the cells were incubated for 36 h. Nuclear extracts were made using Dignam’s protocol [13]. For stable transfection of 32Dcl3 cells the NEPA21 Electroporator was used (NEPA GENE)**.** 32Dcl3 cells and immortalized NUP98-NSD1/FLT3-ITD primary murine cells were lysed using 4X Laemmli buffer, after which the lysate was boiled at 95 °C for 5 min. Patient cells were obtained from the lab of Prof. Olaf Heidenreich at Princes Maxima Center for Pediatric Oncology in Utrecht, The Netherlands. NUP98-NSD1^+^ patient cells were grown in StemSpan™ (STEMCELL Technologies) supplemented with 10 ng/mL IL-3, 10 ng/mL FLT3 ligand, 10 ng/mL GM-CSF, 150 ng/mL SCF, 100 ng/mL TPO.

### Immunoprecipitation

Nuclear extracts were resuspended in IP buffer (10 mM Tris HCL pH 7.6, 150 mM NaCl, 0.4% NP-40, 1X EDTA-free Roche protease inhibitors) to a final concentration of 1 mg/ml. Protein concentration was determined using Bradford assay. FLAG-tagged proteins were immunoprecipitated using anti-FLAG M2-affinity gel (Sigma-Aldrich). For co-immunoprecipitation experiments that were followed by Western blotting, 40 μl of M2 affinity gel was added to 5 mg of nuclear extracts previously resuspended in IP buffer. If the experiment was followed by mass-spectrometry analysis, the same amount of M2 affinity gel was added to 10 mg of nuclear extracts/IP buffer. The reaction was incubated for 10 min at 4 °C, after which the beads were washed two times with “washing buffer-1” (10 mM Tris HCl pH 7.6, 500 mM NaCl, 0.5% NP-40, 1X EDTA-free Roche protease inhibitors), two times with “washing buffer-2” (PBS, 0.5% NP-40, 1X EDTA-free Roche protease inhibitors) and once with PBS. The elution was done using 3XFLAG peptide (#F4799, Sigma-Aldrich) which was resuspended in elution buffer (50 mM Tris HCl pH 7.6, 300 mM NaCl, 1% glycerol,1X EDTA-free Roche protease inhibitors) to a final concentration of 50 ng/ml. Eluted proteins were reduced using 4X Laemmli buffer and boiled at 95 °C.

### Sample preparation and mass spectrometry analysis

Immunoprecipitated samples were run on a 4-12% Bis-Tris Gel (Thermo Scientific) and subjected to Coomassie staining. Protein bands were excised from the SDS-gel. Excised bands were chopped into pieces of approximately 1x1mM. Gel particles were transferred into a 1.5 ml Eppendorf tube where the reduction of proteins’ disulfide bridges was performed using 10 mM DTT in 100 mM NH_4_HCO_3_ at 56 °C for 55 min, while protein alkylation was performed using 55 mM iodoacetamide in 100 mM NH_4_HCO_3_ for 20 min at the room temperature. Proteins were digested with trypsin (0.1 μg/μl in 100 mM NH_4_HCO_3_) and incubated at 37 °C overnight. After digestion, peptides were extracted from the gel pieces using acetonitrile and 5% formic acid. Peptide extracts were then purified using the StageTip procedure [14], dried in a SpeedVac and resuspended in 1% trifluoroacetic acid (TFA) before mass spectrometry analysis. Five microliter of purified peptides were injected into the chromatographic system (Thermo Scientific, EASY-nLC 1200 Liquid Chromatography system), and separated on the self-made capillary column (ReproSil-Pur 120 C18-AQ, 1.9 μm, Dr. Maisch GmbH, 360 × 0.075 mM). Peptide separation was achieved on a linear gradient from 95% solvent A (2% acetonitrile, 0.1% formic acid) to 55% solvent B (80% acetonitrile, 0.1% formic acid) over 75 min and from 55 to 100% solvent B in 3 min at a constant flow rate of 0.25 μl/min on UHPLC Easy-nLC 1000 (Thermo Scientific) where the LC system was connected to a 23-cm fused-silica emitter of 75 μm inner diameter (New Objective, Inc. Woburn, MA, USA), packed in-house with ReproSil-Pur C18-AQ 1.9 μm beads (Dr Maisch Gmbh, Ammerbuch, Germany) using a high-pressure bomb loader (Proxeon, Odense, Denmark).

The LC system was coupled to the Thermo Scientific™ Q Exactive™ HF hybrid quadrupole-Orbitrap mass spectrometer. The total run time including sample loading and column reconditioning was 60 min. The Q Exactive HF was operated in a DDA top 20 method with an MS survey scan resolution setting of 60,000 and MS/MS resolution setting of 17,500. Peptide fragmentation was performed with an NCE (normalized collision energy) of 25 and an isolation window of 2.0 *m/z*. Automatic gain control target value and maximum ion injection times were 3 × 10^5^ and 60 ms for MS, and 10^5^ and 60 ms for MS/MS. Dynamic exclusion was enabled with an exclusion duration of 20s. The mass spectrometry proteomics data have been deposited to the ProteomeXchange Consortium via the PRIDE partner repository with the dataset identifier PXD026020 [15].

### Proteomics analysis

All raw files were processed using MaxQuant [16] (Version 1.5.2.8) against a UniProtKB/Swiss-Prot human database containing 85.336 entries (downloaded on 02.02.2017). Carbamidomethylation was set as fixed modification while methionine oxidation and N-terminal acetylation were searched as variable modifications. Statistical analysis was done in Perseus [17] using two-sample t-tests with Benjamini-Hochberg correction set at FDR = 0.05. Volcano plots were created to display the results of t-testing, with FDR value of 0.05, and S0 value set at 5. For the visualization of this comparison, volcano plots and heatmap were produced using the in-house written R script on which x-axis shows the ratio (fold change) between log2 transformed LFQ values of the proteins bound by NUP98-NSD1 and mutated forms, while on the y-axis -log10 transformed *p* values (obtained in t-test) were plotted.

### ChIP-Seq read alignment

FLAG-NUP98-NSD1 genome binding sites were obtained from a previously published ChiP-seq dataset (GSE112928), expressed in primary murine hematopoietic cells. Reads were aligned to the reference genome (mm10) using bowtie2 algorithm, and peak calling was performed after duplicate filtering using MACS2. To identify NUP98-NSD1 target genes, peaks were intersected with an interval of +/− 1.5 kb around the genomic transcription start sites, which yielded 553 gene targets of the fusion protein. Similarly, SMARCA5 peaks were obtained from the public ChIP-seq dataset acquired in human leukemic K562 cell line (replicates GSM2424122 and GSM2424123), mapped using hg38 human reference genome and intersected with an interval of +/− 1.5 kb around the TSS of the human genome. Acquired SMARCA5 target genes (10,377 genes) were converted into corresponding murine orthologs using an ad-hoc script in R. Gene Set Enrichment Analysis was performed on shared target genes using enrichr package in R assessing the enrichment for the (GO) Biological Processes.

### Immunoblot analysis

Western blotting was performed using standard protocols. Primary antibodies were diluted in 5% milk solution, according to manufacturer’s instructions. We used anti-FLAG (#F7425, Sigma-Aldrich), anti-CHD4 (#ab72418, Abcam), anti-BPTF # A300-973A, Fortis Life Sciences), anti-SMARCA5 (#A301-017A-T, Fortis Life Sciences), anti-KDM1 (#ab37165, Abcam), anti-HDAC2 (#ab12169, Abcam). Primary antibodies were incubated for 2 h at RT with the nitrocellulose membranes. Washing of the nitrocellulose membranes was done 3 times for 5 min, after which secondary antibodies (anti-mouse or anti-rabbit, Bio-Rad) were incubated for 1 h. After three more washes, membranes were developed using the ECL-Plus Western Blotting Reagent (Amersham Biosciences).

### Immunofluorescence

Transfected HEK293 cells were seeded on glass coverslips that were previously placed in 24well plates. 32D cl3, NUP98-NSD1/FLT3-ITD, and patient NUP98-NSD1^+^ cells were attached onto fibronectin-coated glass coverslips (13 mm in diameter). Cells were fixed with 4% paraformaldehyde (Sigma-Aldrich) and permeabilized with 0.2% TritonX-100 (in PBS). Blocking was done using 10% horse serum. Cells were incubated for 1 h at room temperature with primary antibodies, diluted at optimized concentrations in 5% horse serum. Following primary antibodies’ incubation (Anti-FLAG M2 mouse (#F1804, Sigma), 1:2000), the appropriate secondary antibodies were used and incubated with cells for 45 min (Alexa Fluor- coupled antibodies from Jackson ImmunoResearch. Finally, cells were counterstained with DAPI to visualize the nuclei and then mounted on microscope slides using glycerol. The samples were investigated by confocal microscopy performed on a Leica TCS SP5 confocal laser scanning microscope (Leica Microsystem, Heidelberg, Germany). The images were acquired with an HCX PL APO 63X/1.4NA oil immersion objective. The software used for all acquisitions was LAS AF (Leica). Raw images were then analyzed using Fiji software (NIH, Bethesda, USA). The figures were assembled using Adobe Illustrator.

### Proximity ligation assay (PLA)

The Duolink In Situ Far Red Starter Kit Mouse/Rabbit (Sigma-Aldrich) was used for the detection of examined interactions. Cells were attached onto the fibronectin-coated coverslips during overnight incubation at 37 °C with 5% of CO_2_. The following day, the cells were gently washed in PBS and fixed using 4% paraformaldehyde (Sigma-Aldrich). Permeabilization was performed using 0.2% TritonX-100 in PBS at room temperature for 10 min. Then, the cells were gently washed in PBS at room temperature twice. Cells were placed in an in-house made dark and humid chamber. Duolink Blocking solution was used for the blocking step, which was performed at 37 °C for 60 min. Anti-FLAG, SMARCA5 and BPTF antibodies were diluted in Duolink Antibody diluent solution (both in same Eppendorf tube) at the concentration 1:2000, NSD1 (C-term) antibody (#N312/10, NeuroMab) was similarly diluted in Duolink Antibody diluent at the concentration 1:1000, and NUP98 (N-term) antibody (#M1-26400, antibodies-online.com) at the concentration 1:500. After the blocking, incubation with primary antibodies was performed for 60 min at room temperature. Subsequent steps were performed following manufacturer’s instructions. Finally, imaging of these samples was performed on Leica TCS SP5 confocal laser scanning microscope with an HCX PL APO 63X/1.4NA oil immersion objective, and Nikon Crest V3 Spinning Disc Confocal microscope. Images were acquired with LAS AF software (SP5) and NFI software (Nikon) analyzed using Fiji software.

### Fluorescent recovery after photobleaching (FRAP analysis)

FRAP experiments were performed on a Leica TCS SP8 confocal microscope, using HC PL APO CS2 63X/1,40 objective, managed by Leica LasX software. 10 Pre-bleach images were acquired at maximum speed using the white light laser set at 488 nm and 2% power. Then, after bleaching the fluorescence signal at background level using the same laser at maximum power, post-bleach images were acquired according to the following scheme: 30 images at maximum speed, 30 images every 1 s, 50 images every 2 s. Only complete recovery curves were used for the analysis. All of the steps in the analysis were performed using the Leica LasX software, including background subtraction, correction for imaging photobleaching, normalization, curve fitting (single exponential) and mathematical data collection (recovery half-time).

### Biotinylated isoxazole-mediated precipitation

Biotinylated isoxazole (b-isox, Sigma-Aldrich), was resuspended in DMSO. Briefly, HEK293 cells were lysed in lysis buffer (20 mM Tris HCl pH 7.4, 150 mM NaCl, 5 mM MgCl_2_, 0.5% NP-40 and 10% glycerol, supplemented with 1X EDTA-free Roche protease inhibitors for 1 h at 4 °C) [18]. After 30 min centrifugation at maximum speed at 4 °C, protein supernatant was collected, and b-isox was added to 30 μM final concentrations. The reaction was incubated for 1 h at 4 °C. The lysates were then centrifuged for 15 min at 14000 rpm at 4 °C. Pellets were washed twice in lysis buffer and then reduced in 4X Laemmli buffer, while the supernatants were immediately reduced and boiled at 95 °C. Supernatants and pellets were analysed by 4-12% Bis-Tris Gel (Thermo-Scientific) and Western blotting was performed using standard protocols.

### Retroviral transduction of murine hematopoietic stem and progenitor cells

All mouse experiments were in adherence to Swiss animal welfare laws and approved by the Swiss Cantonal Veterinary Office of Basel Stadt (licence no.2087).

For the infection of primary murine HSCs, 6–8 weeks old C57BL/6 mice were primed with 150 mg/kg 5-Fluorouracil (5-FU, Sigma, St Louis, MO) for 6 days. Bone marrow from femurs and tibias was extracted and flushed with the medium using sterile techniques. Bone marrow cells were incubated overnight in RPMI medium containing 10 ng/ml recombinant murine Interleukin-3 (IL-3, Peprotech), 50 ng/ml recombinant murine stem cell factor (SCF, Peprotech), and 10 ng/ml recombinant murine Interleukin-6 (IL-6, Peprotech), 100 U/ml penicillin/streptomycin (Gibco-BRL, Gaithersburg, MD), 20% FBS, 2 mM glutamine, in RPMI 1640 medium (Lonza). Retroviral constructs expressing NUP98-NSD1 *(pMSCVneo-NUP98-NSD1)* and FLT3-ITD-GFP (*pMSCV-FLT3-ITD-IRES-EGFP)* were transiently transfected in Phoenix-ECO cells using calcium phosphate transfection method. Viral supernatants were collected upon 48 h of transfection, filtered using 0.45 μm filters and then concentrated using Vivaspin 20 (Sartorius) columns at 3000 rpm, for 2 h at 4 °C. Concentrated viral supernatant volumes of 200 μl were snap-frozen in liquid nitrogen and kept at − 80 °C. The transduction of 5 × 10^5^ primary murine HSC cells was performed in 2 ml volume (in 12 well plate) comprised of concentrated viral supernatants mixed 1:1 (500 μl each) and 1 ml of RPMI 1640 supplemented with 50 ng/ml SCF, 10 ng/ml IL-3, and 10 ng/ml IL-6 and polybrene at 8 μg/ml. The plates were centrifuged for 90 min at 2500 rpm at 30 °C. Upon centrifugation, the cells were incubated for 4 h, after which 1 ml of RPMI 1640 medium supplemented with IL-3, IL-6, and SCF was added. Transduced cells were expanded in the RPMI1640 medium supplemented with IL-3 (10 ng/ml), IL-6 (5 ng/ml), and SCF (20 ng/ml), and G418 concentration of 0.8 μg/ml.

### Flow cytometry and single-cell sorting

For the GFP sorting and single-cell GFP sorting experiments, cell pellets were washed in PBS, then resuspended in sterile 1% BSA (in PBS) and subjected to FACSAria IIU (Beckton Dickinson) instrument, previously adjusted for corresponding analyses. Doublet events were excluded, while GFP positive singlets were separated from other cells. For the analysis of cell-surface marker expression, cell pellets were washed using 1% BSA (in PBS), blocking was performed in 5% BSA (in PBS) for 45 min at the room temperature. Upon blocking, cell pellets were resuspended in 100 μl of primary antibody solutions, and incubated for 30 min on ice. Then, collected cell pellets were washed two times in 1 ml PBS. Fixation was performed using 1% formaldehyde during 20 min incubation on ice. Finally, the cells were washed once in 1 ml 1%BSA after which the pellets were resuspended in 500 μl of PBS and were subjected to flow cytometry acquisition using Attune NxT (Thermofisher Scientific) instrument. The expression levels of the examined cell surface markers were acquired using FlowJo software.

For flow cytometric analysis, primary human AML cells were washed and suspended in PBS with 1% FBS. Cells were then incubated with a viability reagent, viakrome 808 (Beckman coulter) for 20 min at 4 °C. Then cells washed and centrifuged at 500x g and stained with fluorescence-labelled antibodies against CD14-FITC, CD11b-APC-Cy7, (Biolegend®, San Diego, CA, USA). Flow cytometry experiments were performed on a Beckman Coulter CytoFLEX LX and all data were analyzed with FlowJo software (V10.0.7, TreeStar, Ashland, OR, USA).

### Lentiviral transduction of murine leukemic cells

Lentiviral constructs (3 different shRNAs targeting Smarca5) were transiently transfected in HEK-293 T cells by using the calcium phosphate transfection method together with envelope plasmid *pVSV-G*, and packaging plasmids p*MDL* and *pREV*. Viral supernatants were collected upon 48 h of transfection and filtered using 0.45 μm filter. The transduction of 5 × 10^5^ NUP98-NSD1/FLT3-ITD cells was performed in 2 ml volume (in 6 well plate) comprising viral supernatants mixed with RPMI 1640 supplemented with 20 ng/ml SCF, 10 ng/ml IL-3, and 5 ng/ml IL-6 and polybrene (8 μg/ml). The plates were centrifuged for 90 min at 2500 rpm at 30 °C. Upon centrifugation, the cells were incubated for 4 h, after which 2 ml of RPMI 1640 medium supplemented with IL-3, IL-6, and SCF was added. Day after, the medium was refreshed and the cells were subjected to puromycin antibiotic selection using the concentration of 2 μg/. Upon 4 days, selection medium was removed, and cells that survived were expanded. For the cloning of SMARCA5 shRNAs we used TRCN0000295773 and TRCN0000288446 forward and reverse oligonucleotides, while for the BPTF shRNAs TRCN0000238661 and TRCN0000238664 oligonucleotides were utilized (RNAi Consortium, Broad Institute).

### SMARCA5 knock-down and colony-formation assay

For the induction of Smarca5 knock-down, shRNA transduced cells were plated in RPMI1640 medium supplemented with IL-3, IL-6, SCF, and doxycycline (1 μg/mL). Fresh medium containing doxycycline was continuously exchanged after 48-h-cycles. For colony-forming assays, 15 × 10^3^ shRNA transduced and non-transduced NUP98-NSD1/FLT3-ITD cells were plated in 1.5 mL of methylcellulose (MethoCult M3434, StemCell Technologies) containing IL-3, IL-6, SCF and were grown with/without doxycycline (1 μg/mL). Colonies were scored microscopically after 10 days.

### May-Grunwald Giemsa staining

NUP98-NSD1/FLT3-ITD transduced murine leukemic cells were counted and 2 × 10^5^ cells were spinned at 250 rpm for 5 min. Cytospin slides were dried overnight before the staining procedure. The cells were stained for 8 min with May-Grunwald solution, washed 3 times in deionized water and then incubated for 40 min with Giemsa staining solution. After three more washes with water, samples were dried overnight and the images were acquired using Olympus BX63 upright microscope equipped with Leica DFC450C color camera.

## Results

### NUP98-NSD1 interacts with SMARCA5 and binds its cancer co-dependent partners BPTF and NUP188

**Fig. 1:**
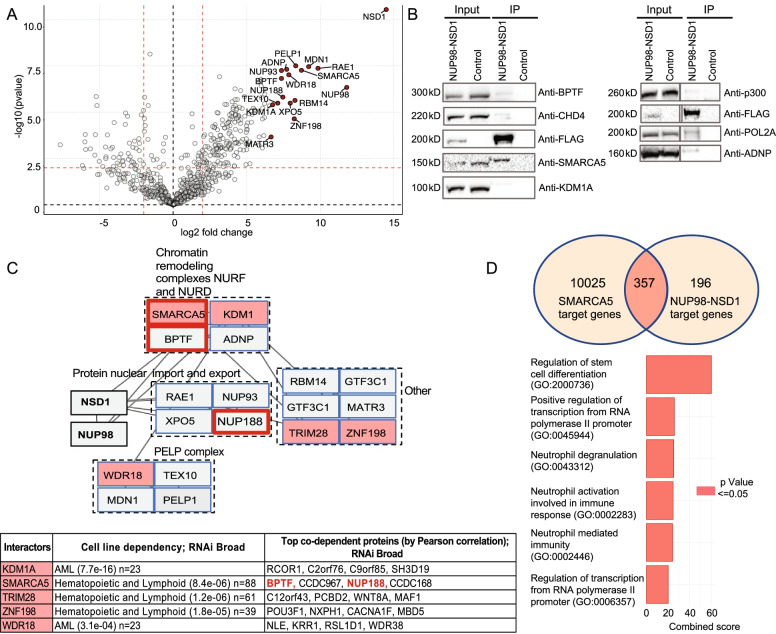
NUP98-NSD1 interacts with SMARCA5 and its cancer co-dependent genes BPTF, and NUP188. **A** Volcano plot showing the enrichment of the bait FLAG-NUP98-NSD1 and the top 20 most enriched interactors (red dots). **B** Western blot validation of NUP98-NSD1 interactions. **C** Cancer dependency analysis of top 20 most enriched interactors. The proteins whose expression is essential for the growth of hematopoietic, blood or acute myeloid leukemia cell lines (KDM1A, SMARCA5, WDR18, ZNF198, TRIM28) are marked in light red. According to Depmap.org, SMARCA5 exhibits co-dependency with BPTF and NUP188 in cancer, while other blood-related top20 interactors do not have cancer-related partners within the core-NUP98-NSD1 interactome. **D** Venn diagram shows the overlap between NUP98-NSD1 and SMARCA5 gene targets. The plot indicates the most represented biological processes belonging to common gene targets

Due to the absence of patient-derived NUP98-NSD1^+^ AML cell lines and limited access to primary tumor cells we determined protein-protein interactions of NUP98-NSD1 in human embryonic kidney (HEK-293) cells. We transiently expressed FLAG-NUP98-NSD1 (control cells were transfected with empty FLAG vector) and prepared nuclear lysates for immunoprecipitation (IP) followed by mass spectrometry. Protein label-free quantification (LFQ) values from two replicate samples were analyzed using two-samples t-test statistics (Benjamini-Hochberg correction with FDR = 0.05) leading to the quantification of 502 proteins out of which 182 were statistically significant potential NUP98-NSD1 interactors (Supplementary Table 1). Importantly, the previously validated NUP98-NSD1 interactors RAE1 and XPO5 [19] were among the most significantly enriched proteins (Fig. 1A). Another previously known interactor, the EP300 histone acetyl-transferase [20], was validated by Western-blotting (Fig. 1B). In addition, the interactions with SMARCA5, BPTF, ADNP, KDM1A, CHD4 and POL2A were also verified by Western-blotting (Fig. 1B). To get mechanistic insights, potential functional co-operation among top 20 most enriched interactions was examined using the Cancer Dependency Map (Depmap) which revealed that SMARCA5, BPTF and NUP188 operate as a co-dependent functional unit in cancer cells (Fig. 1C). To determine the potential functional relations between NUP98-NSD1 and SMARCA5 we examined their genomic binding in leukemic cells using public ChIP-Seq datasets. Peak enrichment analysis showed that NUP98-NSD1 co-localized with SMARCA5 at 65% of its target sites. Moreover, Gene Set Enrichment Analysis (GSEA) revealed that co-bound genes are involved in regulation of stem cell differentiation and positive regulation of transcription from RNA II polymerase promoter (Fig. 1D). Finally, a separate analysis of the interactomes of WT NSD1 and the NUP98 N-terminus uncovered that SMARCA5 was the only protein among the 20 most enriched interactors, to be bound independently by WT-NSD1 and N-terminal NUP98, which suggests a deeply rooted molecular connection with the fusion protein (Supplementary Fig. 1 & Tables 2 and 3). Taken together, systematic analysis of NUP98-NSD1-bound proteome identified the ISWI family protein SMARCA5 (and its cancer co-dependent partners BPTF and NUP188) as stable interactors and revealed their potential functional co-operation in transcriptional regulation of stem cell differentiation.

### NUP98-NSD1 core interactome binding relies on NUP98 FG repeat domains

**Fig. 2:**
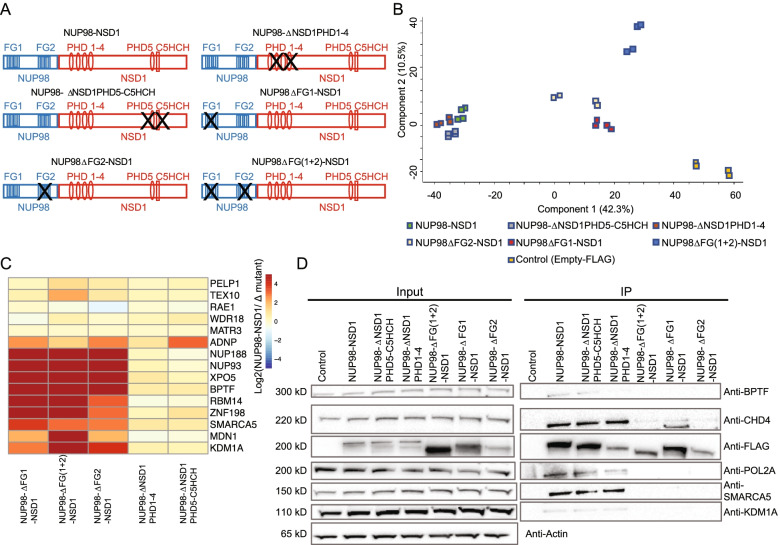
NUP98-NSD1 interactome binding relies on disordered FG repeat domains. **A** Scheme displaying deletion of specific NUP98-NSD1 domains. **B** Principal component analysis assessing the level of variation between the interactor abundances (LFQ values) within the full-length and mutated fusion interactomes. **C** Heatmap shows the log2 fold change (log2 transformed ratio between the LFQ values of the interactors bound by NUP98-NSD1 vs the mutated constructs) of top20 most confident NUP98-NSD1 interactors upon specific domain deletions in NUP98-NSD1. **D** Co-immunoprecipitation coupled to Western blot validation of lost interactions upon deletion of single or both FG repeat domains

To examine the role of different NUP98-NSD1 domains in interactome organization, we generated NUP98-NSD1 mutants bearing deletions of one or both FG repeat motifs (FLAG-ΔNUP98FG1-NSD1, FLAG-ΔNUP98FG2-NSD1, FLAG-ΔNUP98FG(1 + 2)-NSD1), of PHD1-4 domains (FLAG-NUP98-ΔNSD1PHD1-4), and of PHD5 and C5HCH domains (FLAG-NUP98-ΔNSD1PHD5-C5HCH) and transiently expressed them in HEK-293 cells (Fig. 2A). We achieved almost equilibrated expression and immunoprecipitation of the mutated constructs (Fig. 2D, Supplementary Fig. 2). Interactors of full-length NUP98-NSD1 and of its mutated versions were identified by label free quantitative proteomics. Strikingly, principal component analysis (PCA) separated the interactomes acquired for the FG-repeat motif deleted fusions and control on the one side, and full-length and PHD1-4 and PHD5-C5HCH deleted fusions on the other side, suggesting a prominent role of the disordered domains in the interactome organization (Fig. 2B-D, Supplementary Fig. 3). To assess the impact of the mutated domains on the core interactome, we analyzed the LFQ values of the top 20 NUP98-NSD1 interactors and observed significant perturbations in this core interactome upon deletions of single or both FG repeat domains, whereas deletions of the PHD domains barely affected these interactions (Fig. 2C, Supplementary Fig. 3, Supplementary Tables 4-6). In particular, SMARCA5, BPTF and NUP188 were lost upon deletion of one or both FG repeat domains. In addition, interactions with KDM1, NUP93, XPO5, RBM14, or MDN1 were also evicted in absence of one or both FG repeats (Fig. 2C). As expected, the interaction with RAE1, mediated by the GLEBS domain of NUP98 moiety, was not affected by FG repeat deletions (Fig. 2C, Supplementary Fig. 3B), indicating that our analysis identified specific, FG-repeat mediated interactions. Loss of interaction with BPTF, SMARCA5, KDM1A, CHD4 and POL2A was confirmed by Western blotting (Fig. 2D). Taken together, mutational studies revealed a prevalent role of the NUP98 FG repeat domains in mediating the interactome network of NUP98-NSD1.

### NUP98 FG repeat domains mediate the formation of NUP98-NSD1 liquid-like phase separated nuclear condensates

**Fig. 3:**
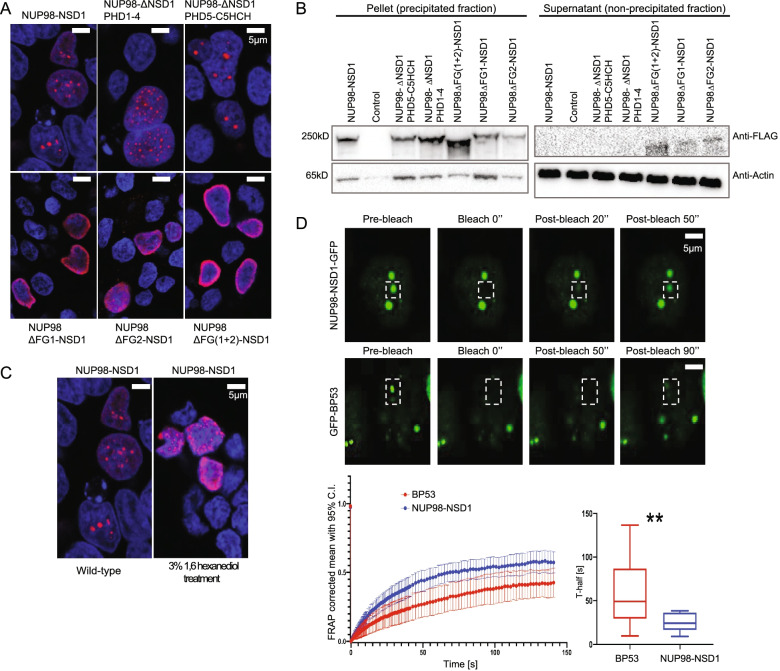
NUP98 FG repeat domains mediate the formation of NUP98-NSD1 liquid-like phase separated nuclear condensates. **A** Immunofluorescence staining of HEK293 cells transiently overexpressing full-length NUP98-NSD1 and its mutated forms. The cells were stained with Anti-FLAG antibody labeled with Alexa Fluor 568 secondary antibody (red). Nuclei were counterstained with DAPI (blue). **B** Precipitation of NUP98-NSD1 and its mutated forms in the presence of 30 μM b-isoxazole. Deletion of one or both FG repeat domains causes a decrease in the phase transition capacity of the fusion protein, represented by the signal in the supernatant. **C** Immunofluorescence analysis of HEK293 cells upon the treatment with 3% 1,6 hexanediol. The cells were stained with Anti-FLAG antibody labeled with Alexa Fluor 568 secondary antibody (red). Nuclei were counterstained with DAPI (blue). NUP98-NSD1 nuclear puncta were dispersed upon the treatment. **D)** Representative images of FRAP experiment of NUP98-NSD1-GFP and GFP-BP53 expressing HEK293 cells, with white boxes highlighting the punctum undergoing targeted bleaching. Fluorescence recovery over time was followed and quantified for NUP98-NSD1-GFP and GFP-BP53 (lower left panel). T-test comparison of the acquired fluorescence recovery half-times (T-half) was performed showing statistically significant faster recovery half-time for NUP98-NSD1-GFP. **P < 0.01, *N* = 54

To examine the nuclear localization pattern of NUP98-NSD1 and the dependency on its structural domains, we performed immunofluorescence staining of transiently expressed full-length and mutated NUP98-NSD1 constructs. Confocal imaging showed that the full-length NUP98-NSD1 displayed a “nuclear puncta” distribution, dependent on both FG-repeat domains as their deletion led to complete dispersion and to a more peripheral localization of the fusion protein (Fig. 3A). On the other side, deletion of PHD1-4 and PHD5-C5HCH domains did not significantly affect the formation of nuclear puncta, suggesting that FG repeat domains are pivotal for the correct nuclear localization of NUP98-NSD1 (Fig. 3A). As these disordered domains mediate phase separation of the full-length NUP98 within the NPC [21], we examined whether NUP98-NSD1 exhibits phase-separating properties. To assess the phase-transition potential of NUP98-NSD1 in vitro, we tested its ability to precipitate in presence of biotinylated isoxazole, a compound that induces the precipitation of proteins with disordered domains [18]. While the full-length protein and the PHD-deleted forms achieved complete precipitation with 30 μM b-isoxazole, the FG-deleted constructs exhibited a “leakage” in the unprecipitated fraction (Fig. 3B), suggesting that FG repeat domains are indeed required for phase transition of NUP98-NSD1. In addition, NUP98-NSD1 nuclear puncta were sensitive to 1,6 hexanediol treatment, a compound that disrupts the formation of liquid-liquid like biomolecular condensates [22], thus confirming their phase-separated nature (Fig. 3C). To analyze the properties of NUP98-NSD1 nuclear condensates in vivo, we transiently expressed a NUP98-NSD1-GFP fusion and performed FRAP analysis to study the dynamics of the protein in live cells. As positive control, HEK293 cells were transfected with GFP-BP53, known to exhibit liquid-like phase separation in live cells [23]. After photobleaching, NUP98-NSD1-GFP nuclear puncta recovered fluorescence on a time-scale of ~ 50 s, and exhibited significantly faster motility than GFP-BP53 which recovered fluorescence after ~ 90 s, thus demonstrating the liquid-like nature of phase-separated NUP98-NSD1 nuclear puncta (Fig. 3D). Taken together, our data suggest that NUP98-NSD1 forms liquid-like phase-separated nuclear condensates dependent on NUP98 FG repeat domains. Moreover, in line with proteomics data, the identified FG-repeat mediated interactors are bona fide constituents of NUP98-NSD1 condensates.

### NUP98-NSD1 interactions and condensate formation are conserved in NUP98-NSD1/FLT3-ITD immortalized hematopoietic cells

**Fig. 4:**
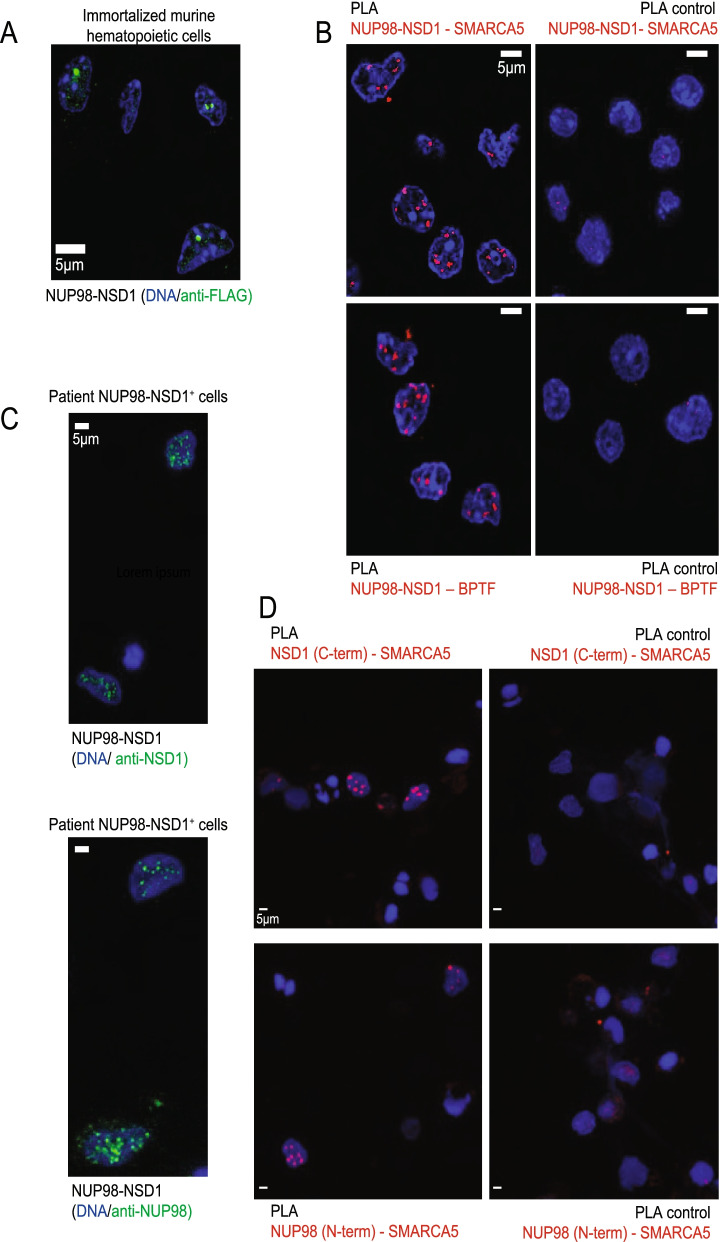
NUP98-NSD1 retains nuclear localization pattern and interactions in murine NUP98-NSD1/FLT3-ITD immortalized hematopoietic stem and progenitor cells and in NUP98-NSD1^+^ patient cells. **A** Immunofluorescence staining showing the nuclear puncta pattern of localization of the fusion in primary murine NUP98-NSD1/FLT3-ITD immortalized hematopoietic cells. The cells were stained with Anti-FLAG antibody labeled with Alexa Fluor 568 secondary antibody (green). Nuclei were counterstained with DAPI (blue). **B** Proximity ligation assay in NUP98-NSD1/FLT3-ITD immortalized hematopoietic cells showing the interaction between NUP98-NSD1 and SMARCA5/BPTF, detected using kit specific fluorophore (lem = 669 nm, far red). Nuclei were counterstained with DAPI (blue). **C** Immunofluorescence staining showing the nuclear puncta pattern of localization of the fusion in NUP98-NSD1^+^ patient cells. The cells were stained with Anti-NSD1 and Anti-NUP98 antibodies targeting the C-terminus and N-terminus (epitopes included in the fusion protein), respectively. Primary antibodies were then labeled with Alexa Fluor 647 secondary antibody (green). Nuclei were counterstained with DAPI (blue). **D** Proximity ligation assay in NUP98-NSD1^+^ patient cells showing the interaction between the N-terminal NUP98 and C-terminal NSD1 moieties of the fusion with SMARCA5, detected using kit specific fluorophore (lem = 669 nm, far red), on the right. Nuclei were counterstained with DAPI (blue)

To assess whether the phase-separated nature of NUP98-NSD1 is conserved in hematopoietic cells, we expressed the full-length NUP98-NSD1 and ΔNUP98FG(1 + 2)-NSD1 in 32Dcl3 murine myeloid progenitor cells. Importantly, the fusion protein kept the nuclear puncta localization pattern, which was dependent on the presence of FG repeats (Supplementary Fig. 4). To study the functional impact of our findings on NUP98-NSD1-mediated transformation, we immortalized primary murine bone marrow cells by retroviral expression of NUP98-NSD1 and FLT3-ITD, previously shown to be necessary for efficient immortalization of primary murine hematopoietic stem and progenitor cells [24]. After expansion under antibiotic selection and enrichment by GFP sorting (Supplementary Fig. 5A), we confirmed the presence of NUP98-NSD1 nuclear puncta by immunofluorescence staining (Fig. 4A). Giemsa-Wright staining revealed a blast-like morphology of NUP98-NSD1/FLT3-ITD cells (Supplementary Fig. 5B). To examine whether the fusion protein interacts with SMARCA5 and BPTF also in these cells, we performed proximity ligation assays. We found that the signal for both interactions was specifically located in the nucleus of NUP98-NSD1/FLT3-ITD cells, while control samples showed low background (Fig. 4B). To address the clinical relevance of our findings, we examined nuclear localization pattern and tested the observed interactions in NUP98-NSD1^+^ patient cells, using the antibodies targeting N-terminal NUP98 and C-terminal NSD1. Immunofluorescence staining of both moieties showed punctual pattern of nuclear localization (Fig. 4C), which was in line with recently published findings in patient cells expressing NUP98-HOXA9, NUP98- PRRX1, NUP98-KDM5A, and NUP98-LNP1 fusions [5]. Moreover, we detected the interaction between SMARCA5 and both fusion moieties in NUP98-NSD1^+^ patient cells, thus confirming the existence of this interaction in physiological conditions (Fig. 4D). Overall, these data indicate that the NUP98-NSD1 interactome and nuclear localization identified in HEK-293 cells are present in NUP98-NSD1 expressing patient cells and bona fide maintained in immortalized primary murine hematopoietic cells.

### SMARCA5 is indispensable for NUP98-NSD1-mediated transformation and expression of the fusion target genes

**Fig. 5:**
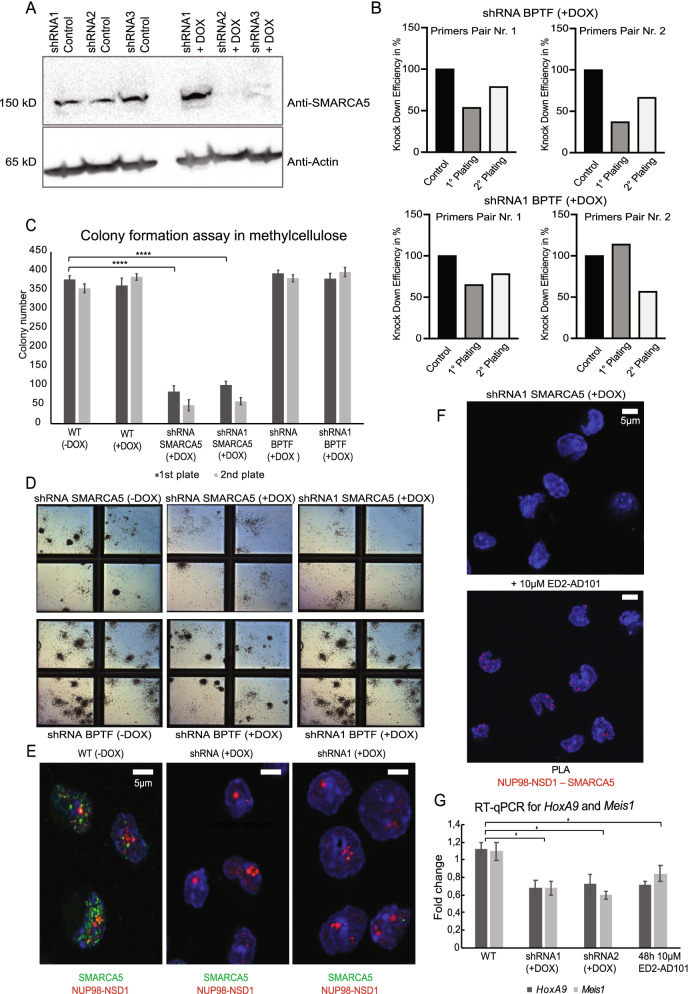
SMARCA5 genetic and pharmacologic targeting reduce self-renewal of NUP98-NSD1/FLT3-ITD immortalized hematopoietic stem and progenitor cells and downregulate *HoxA9* and *Meis1* expression. **A** Western-blot analysis showing the expression of SMARCA5 in NUP98-NSD1/FLT3-ITD hematopoietic cells. **B** RT-qPCR analysis showing the knockdown of BPTF at mRNA level, upon 1st and 2nd methylcellulose (re)plating using 2 different primers for 2 different shRNAs. **C** Colony numbers after (re)plating in methylcellulose upon treatment with 2 μg/ml doxycycline. *N* = 3 biological replicates. T-test, ****P < 0.0005. Data are mean ± SD. **D** Colony formation capacity **in** methylcellulose after plating in presence of 2 μg/ml doxycycline. The images represent the effect observed in 3 biological replicates. **E** Immunofluorescence staining of NUP98-NSD1/FLT3-ITD immortalized hematopoietic cells upon doxycycline induced knockdown of SMARCA5, showing formation of the fusion’s nuclear condensates irrespectively from SMARCA5 expression. The cells were stained using Anti-FLAG and Anti-SMARCA5 antibodies, labeled with Alexa Fluor 568 secondary antibody (red), and Alexa Fluor 647 secondary antibodies (green), while nuclei were counterstained with DAPI (blue). **F** Proximity ligation assay in NUP98-NSD1/FLT3-ITD immortalized hematopoietic cells showing the lack of the signal for the interaction between NUP98-NSD1 and SMARCA5 in shRNA1 + DOX (SMARCA5 knockdown) condition and upon treatment with 10 μM ED2-AD101. **G** *HoxA9* and *Meis1* expression measured by RT-qPCR in cells upon induced shRNA-mediated knockdown of SMARCA5 (1 μg/ml doxycycline) and treatment with 10 μM ED2-AD101. N = 3 biological replicates. T-test, *P < 0.05. Data are mean ± SD

To examine the functional role of SMARCA5 and BPTF interactors in NUP98-NSD1/FLT3-ITD immortalized hematopoietic cells, we performed doxycycline-inducible (DOX) knock-down of both nuclear condensate constituents. We confirmed the knockdown of SMARCA5 at protein and of BPTF at the mRNA level (Fig. 5A, B), and analyzed the clonogenic growth of NUP98-NSD1/FLT3-ITD transformed cells in methylcellulose. Although SMARCA5 and BPTF proteins cooperate within the NURF chromatin remodeling complex, the transformed cells were specifically sensitive to SMARCA5 loss (Fig. 5C), which completely abolished colony formation (Fig. 5D) thus indicating its functional role in mediating NUP98-NSD1 transformation. Interestingly, the knockdown of SMARCA5 did not influence formation of fusion phase separated nuclear condensates (Fig. 5E), despite recently reported critical role of biomolecular condensation in NUP98 leukemogenesis, suggesting the importance of fully functional interactome network in the maintenance of NUP98-NSD1 transformation. To further examine SMARCA5 function in transformed cells, we performed treatment with an allosteric inhibitor (ED2-AD101) targeting the ATPase activity of the ISWI family of chromatin remodelers (Supplementary Fig. 5) [25, 26], which consistently prevented colony formation in methylcellulose (Supplementary Fig. 6). NUP98-NSD1 requires catalytically active SMARCA5 for the maintenance of hematopoietic transformation, as the interaction remained unaffected upon pharmacological inhibition (Fig. 5F). Finally, SMARCA5 genetic and pharmacological targeting led to reduced expression of NUP98-NSD1 gene targets *HoxA9* and *Meis1*, which proposes its role in regulation NUP98-NSD1-driven transcriptional program (Fig. 5G). To further address the clinical relevance of ED2-AD101, we treated NUP98-NSD1^+^ patient cells using 10 μM concentration (following the previously calculated IC_50_ in transformed murine hematopoietic cells, Supplementary Fig. 6) and found some increase in expression of the CD11b and CD14 surface markers of myeloid differentiation (Supplementary Fig. 7A). However, due to high cell death (Supplementary Fig. 7B) upon inhibitor treatment we could not characterize the exact effect of the inhibitor, probably caused by the low specificity of the compound. In summary, our genetic and pharmacologic data in transformed murine and patient cells suggest a pivotal role of SMARCA5, constituent of the NUP98-NSD1 nuclear condensates, in the maintenance of NUP98-NSD1-mediated transformation.

## Discussion

We characterized the nuclear core interactome of the AML-associated NUP98-NSD1 fusion protein and identified the constituents of the NUP98 phenylalanine-glycine (FG) repeat-dependent nuclear condensates. We also found functional co-dependencies of condensate constituents including the transcriptional coregulators SMARCA5 and BTPF. Genetic and pharmacological interference characterized SMARCA5 as NUP98-NSD1 interacting protein critical for aberrant clonogenic growth of NUP98-NSD1 immortalized primary murine hematopoietic cells. Notably the interaction of NUP98-NSD1 and SMARCA5 was also found in primary patient-derived AML cells carrying this fusion.

Several very recent studies proposed that FG repeat-mediated biomolecular condensation of NUP98 fusions is crucial and sufficient to activate a leukemogenic transcriptional program [4–6]. However, none of the studies examined any molecular players that cooperate with NUP98 fusions in installing the leukemogenic gene regulatory network.

In our study, we demonstrated that both genetic and pharmacological targeting of SMARCA5 did not affect the condensates indicating that biomolecular condensation per se is not sufficient to maintain the transformed phenotype. One recent study compared the protein interactome of five AML-associated epitope-tagged NUP98 fusions, including NUP98-NSD1, ectopically expressed in the human HL-60 AML cell line and identified by mass spectrometry 157 shared interactors [6]. Notably, in contrast to our study, Terlecki-Zaniewicz and colleagues identified SMARCA5 (also known as SNF2H) as general constituent of biomolecular condensates in human HL-60 AML cells, but not as interactor of any of the 5 NUP98-fusions studied. We believe that the reason for the observed discrepancy lies in diverse experimental approaches linked to different objectives of this recent publication. Terlecki-Zaniewicz and colleagues performed a very challenging screen for common interactions among 5 different NUP98 fusions using whole cell lysates. On the other hand, we used nuclear extracts in our mass spectrometry and Western blot analysis (as NUP98-NSD1 is expressed exclusively in the nucleus), in order to reduce the number of cytoplasmic contaminants. Furthermore, we focused on dissecting in-depth the nuclear interactome of solely NUP98-NSD1, and therefore utilized an immunoprecipitation protocol enabling the detection of high-confidence protein-protein interactions [27]. Moreover, we provided specific functional information related to the dissection of NUP98-NSD1 nuclear condensate constituents through structural domain-dependent interactome analysis. Finally, we also validated some interactions in NUP98-NSD1 immortalized primary murine hematopoietic cells and patient cells using proximity ligation assay.

Another recent study demonstrated that altered chromatin looping, induced by the phase separation of NUP98-HOXA9, is driving the onset of the leukemogenic transcription programs [4]. Protein-mediated phase separation contributes to the three-dimensional organization of the genome and transcription regulation, thus influencing the expression of gene regulatory networks driving the cell fate [28]. SMARCA5 chromatin remodeler is one of the principal regulators of genome-wide nuclear topology, and is deeply involved in recruitment of transcription factors, such as CTCF [29]. We showed that genomic co-localization of SMARCA5 and NUP98-NSD1 occurs at the transcriptionally active genomics sites regulating stem cell differentiation, therefore suggesting that SMARCA5 may recruit NUP98-NSD1 to its target sites, thus promoting leukemic transformation.

Genetic ablation of *Smarca5* in mice leads to an early lethal phenotype due to blocked maturation of erythroid and myeloid lineages underlining its role as a critical hematopoietic regulator [30]. In hematopoiesis, highest SMARCA5 levels are expressed in progenitor cells of the myeloid and erythroid lineage (http://servers.binf.ku.dk/bloodspot/). Likewise, exposure to a small molecule inhibitor (ED2-AD101) targeting the chromatin remodelers SMARCA5 and CHD4 at doses of between 1 to 10 μM induced differentiation of THP-1 leukemic cell line [25]. We observed some degree of differentiation of NUP98-NSD1^+^ patient cells upon treatment with 10 μM ED2-AD101, and very high rate of cell death, most probably due to the low specificity of the compound. Therefore, more selective SMARCA5-inhibiting compounds are clearly necessary to explore a therapeutic window against AML.

We, as well as Terlecki-Zaniewicz and colleagues, did not identify the previously published interaction of NUP98-fusions with KMT2A detected using whole-cell lysates and proximity dependent biotinylation (BioID) followed by Western blotting [11]. This may be explained by the biochemical complementarity of the different approaches [31, 32]. We also compared our NUP98-NSD1 interactome with the affinity-purified NUP98-HOXA9 interactome reported by Shima et al. and found a rather low overlap, suggesting that two fusion proteins may utilize different mechanisms for their molecular function [33]. Nevertheless, both datasets shared previously published NUP98 interactions with RAE1 and XPO1.

## Conclusions

In conclusion, we characterized multiple nuclear protein interactions of the AML-associated NUP98-NSD1 fusion and identified SMARCA5 as important mediator to maintain the transformed phenotype of NUP98-NSD1-immortalized hematopoietic cells. Our data contain thorough functional information that may be crucial for designing specific inhibitors to be used in NUP98-NSD1 driven AML.

## Supplementary Information

**Additional file 1.**

## Data Availability

The mass spectrometry proteomics data have been deposited to the ProteomeXchange Consortium via the PRIDE partner repository with the dataset identifier PXD026020.

## Abbreviations

AML: Acute myeloid leukemia
FG: Phenylalanine-glycine
ISWI: Imitation switch family of proteins
BPTF: Bromodomain PHD finger transcription factor
NURF: Nucleosome remodeling factor
NUP98: Nucleoporin 98
NSD1: Nuclear receptor SET domain
PHD: Plant homeodomain
C5HCH: Cys-His-rich
PWWP: Proline-tryptophan-proline
ATCC: American Type Culture Collection
TFA: Trifluoroacetic acid
PLA: Proximity ligation assay
FRAP: Fluorescent recovery after photobleaching
b-isox: b-isoxazole
5-FU: 5-Fluorouracil
IL-3: Interleukin 3
IL-6: Interleukin 6
SCF: Stem cell factor
HEK293: Human embryonic kidney cells
IP: Immunoprecipitation
LFQ: Label-free quantification
GSEA: Gene set enrichment analysis
PCA: Principal component analysis
DOX: Doxycycline
shRNA: Small hairpin RNA
BioID: Proximity dependent biotinylation
