# Supplementary material for: SMARCA5 interacts with NUP98-NSD1 oncofusion protein and sustains hematopoietic cells transformation
Source: J Exp Clin Cancer Res. 2022 Jan 24;41:34. doi: 10.1186/s13046-022-02248-x (PMC8785526; doi:10.1186/s13046-022-02248-x)
Supplement: Supplementary file 1 — Additional file 1. [file 13046_2022_2248_MOESM1_ESM.pdf]

SUPPLEMENTARY INFORMATION

SMARCA5 interacts with NUP98-NSD1 oncofusion protein and sustains hematopoietic cells transformation

Zivojin Jevtic[1], Vittoria Matafora[1], Francesca Casagrande[2], Fabio Santoro[3], Saverio Minucci[3,4], Massimilliano Garre'[2], Milad Rasouli[5], Olaf Heidenreich[5], Giovanna Musco[6], Jürg Schwaller[7*], Angela Bachi[1*]

1 Functional Proteomics group, IFOM-FIRC Institute of Molecular Oncology, Milan, Italy

2 Imaging technological development unit, IFOM-FIRC Institute of Molecular Oncology, Milan, Italy

3 Chromatin Alterations in Tumorigenesis unit, European Institute of Oncology, Milan, Italy

4 Department of Biosciences, University of Milan, Milan, Italy

5 Princes Maxima Center for Pediatric Oncology, Utrecht, The Netherlands

6 Biomolecular NMR, IRCCS Ospedale San Raffaele, Milan, Italy

7 Department for Biomedicine, University Children Hospital, Basel, Switzerland

*Corresponding author: angela.bachi@ifom.eu, j.schwaller@unibas.ch

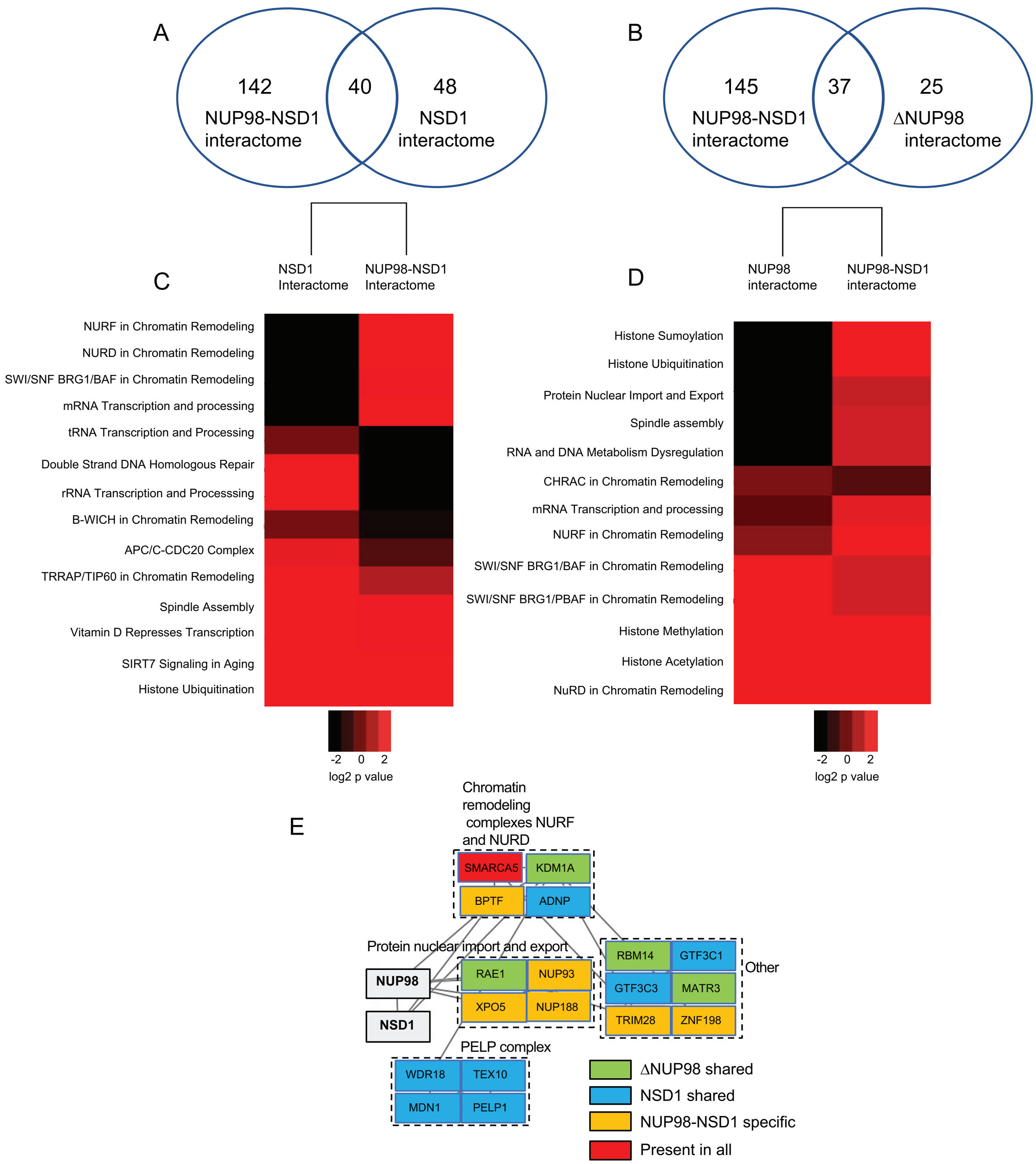

Supplementary Figure 1. Comparison of NUP98-NSD1 interactome with the WT-NSD1 and ΔNUP98 interactomes: **A)** Venn diagram showing the comparison of statistically significant FLAG-NSD1 and FLAG-NUP98-NSD1 interactors obtained in corresponding analyses. **B)** Venn diagram showing the comparison of statistically significant FLAG-ΔNUP98 and FLAG-NUP98-NSD1 interactors obtained in

corresponding analyses. **C,D)** Compared functional annotations for FLAG-NSD1, FLAG-∆NUP98 and FLAG-NUP98-NSD1 interactomes. Most confident annotations from the KEGG pathway collection (based on the p values) were compared for the three analyses. **E)** Summary diagram of top 20 most confident NUP98-NSD1 interactors (taken from the Figure 19) with the color-code displaying the interactions shared with NSD1 and ∆NUP98, specific for NUP98-NSD1, and those that were scored in all three interactome analyses.

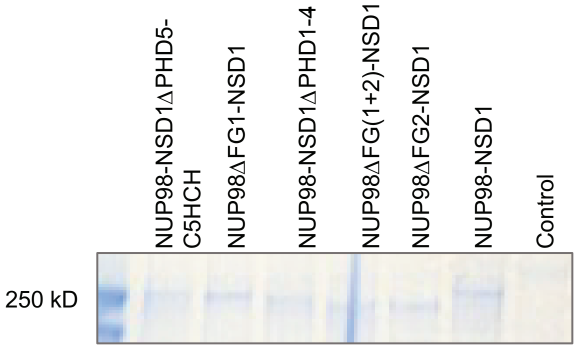

Supplementary Figure 2. Equilibrated immunoprecipitation of FLAG-NUP98-NSD1 mutated forms in HEK293 cells. Representative SDS-PAGE gel stained by Coomassie staining showing immunoprecipitation of the fusion protein and its mutated forms.

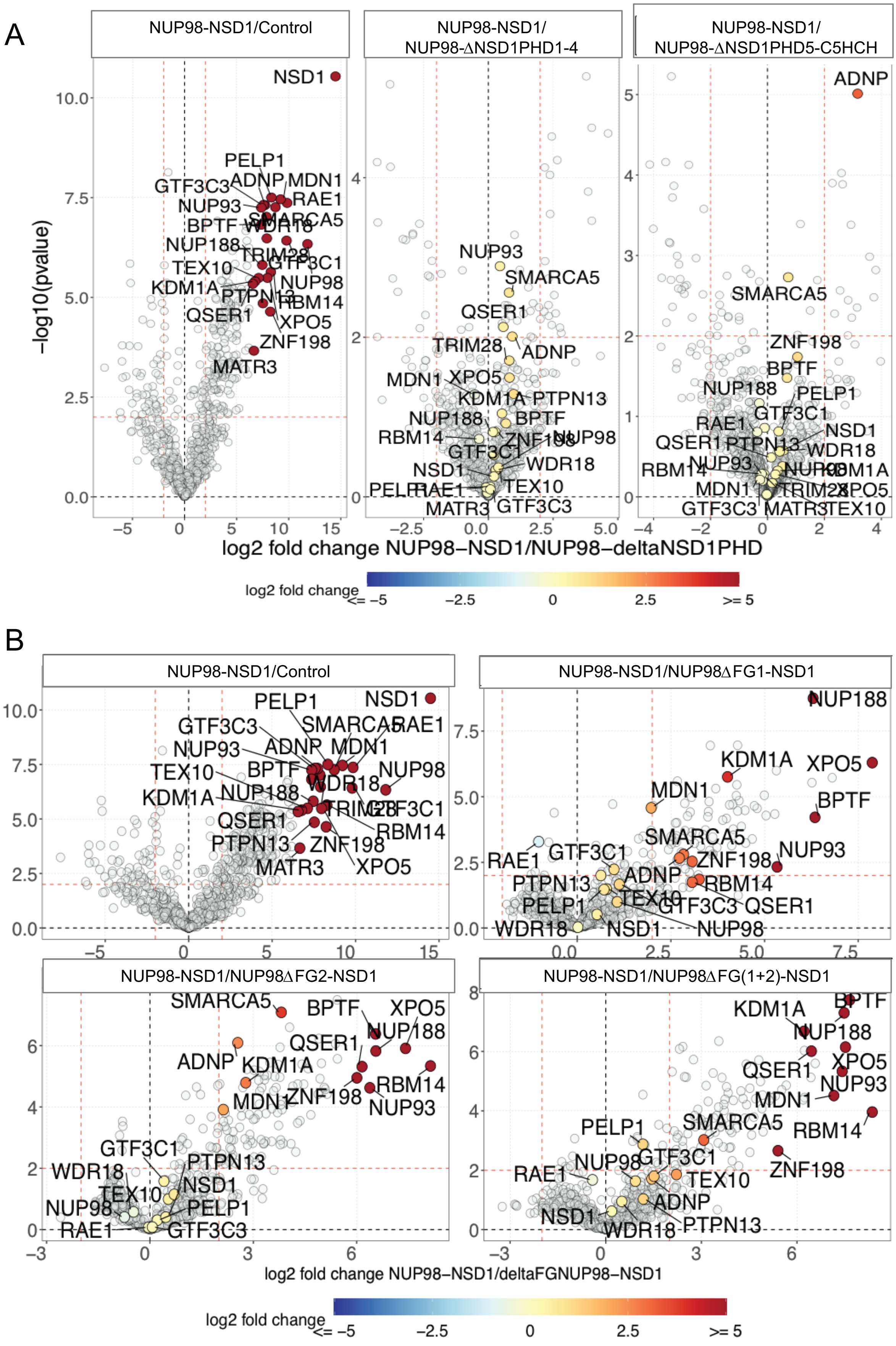

Supplementary Figure 3. Domain specific analysis of core NUP98-NSD1 interactors. **A,B)** Volcano plots demonstrating statistically significant alterations of

NUP98-NSD1 interactome upon the PHD1-4, PHD5, FG1, FG2, and FG(1+-2) deletions. Labeled data points present top20 most confident interactors of NUP98-NSD1. On x-axis, the ratio between log2 transformed LFQ values of the proteins bound by NUP98-NSD1 and NUP98-ΔNSD1PHD5 or NUP98-ΔNSD1PHD1-4 (log2 fold change) was plotted and on the y-axis the -log10 transformed p-values obtained from t-test comparison.

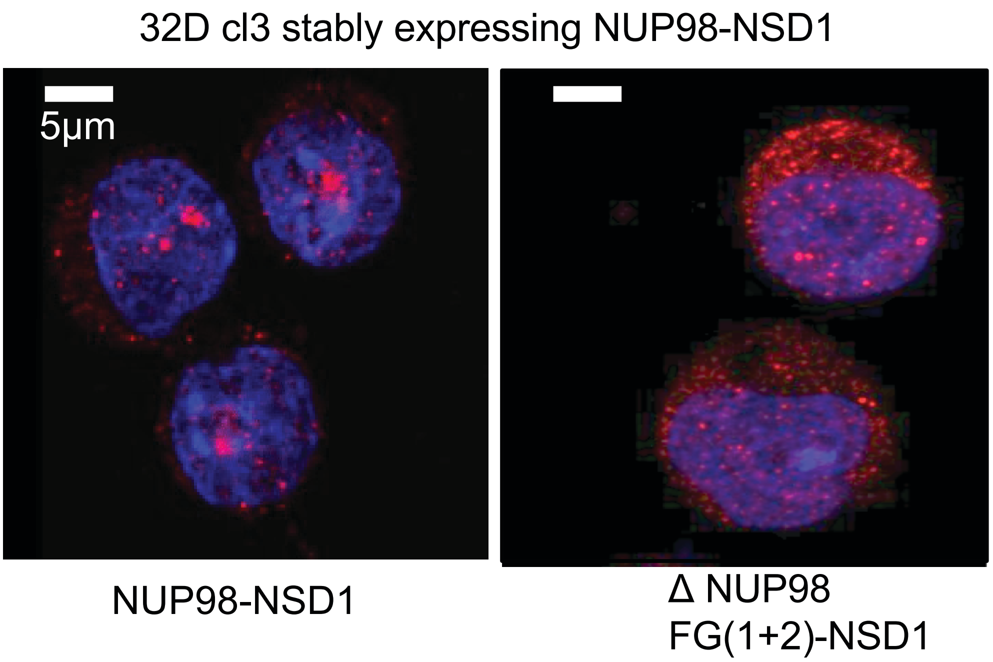

Supplementary Figure 4. Immunofluorescence staining of 32D cl3 cells stably expressing FLAG-NUP98-NSD1 and FLAG-ΔNUP98FG(1+2)-NSD1 showing loss of nuclear condensate formation upon deletion of FG repeat domains. The cells were stained with Anti-FLAG antibody labeled with Alexa Fluor 568 secondary antibody (red). Nuclei were counterstained with DAPI (blue).

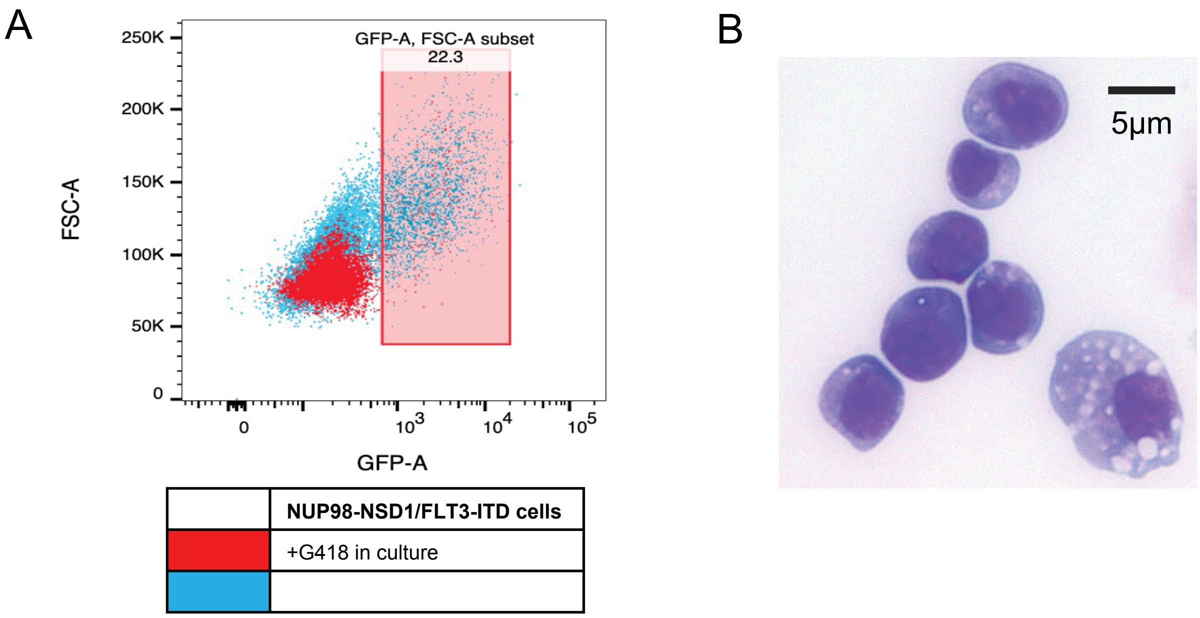

Supplementary Figure 5. Generation and characterization of the NUP98-NSD1/FLT3-ITD immortalized primary murine myeloid cell line. A) Representative FACS plots showing the percentage of GFP+ cells (expressing FLT3-ITD). Control sample (cells grown without antibiotic selection) were colored in red, while cells expressing FLT3-ITD-GFP were colored in blue. B) Giemsa-wright staining showing the blast morphology of NUP98-NSD1/FLT3-ITD cells.

A

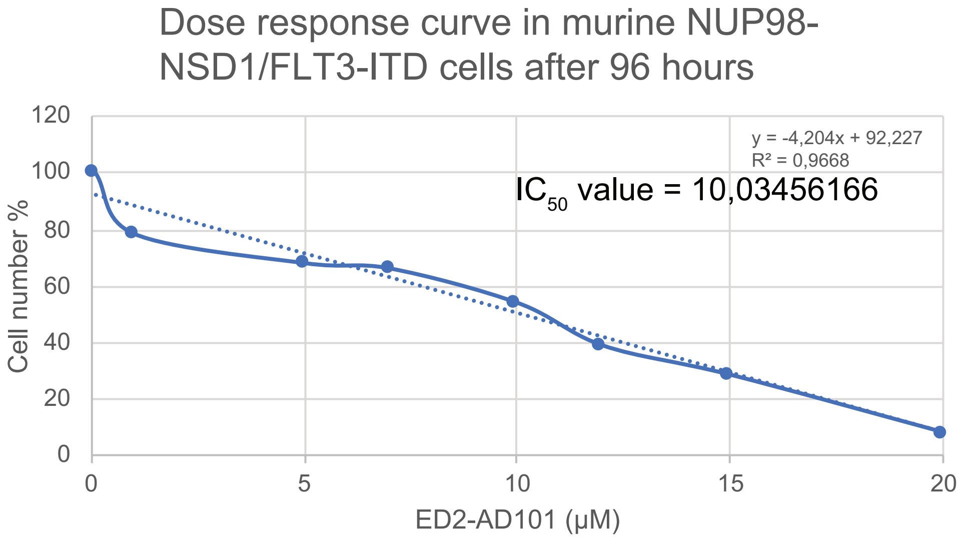
Dose response curve in murine NUP98-NSD1/FLT3-ITD cells after 96 hours
y = -4,204x + 92,227
R² = 0,9668
IC50 value = 10,03456166
Cell number %
120
100
80
60
40
20
0
0
5
10
15
20
ED2-AD101 (µM)

B

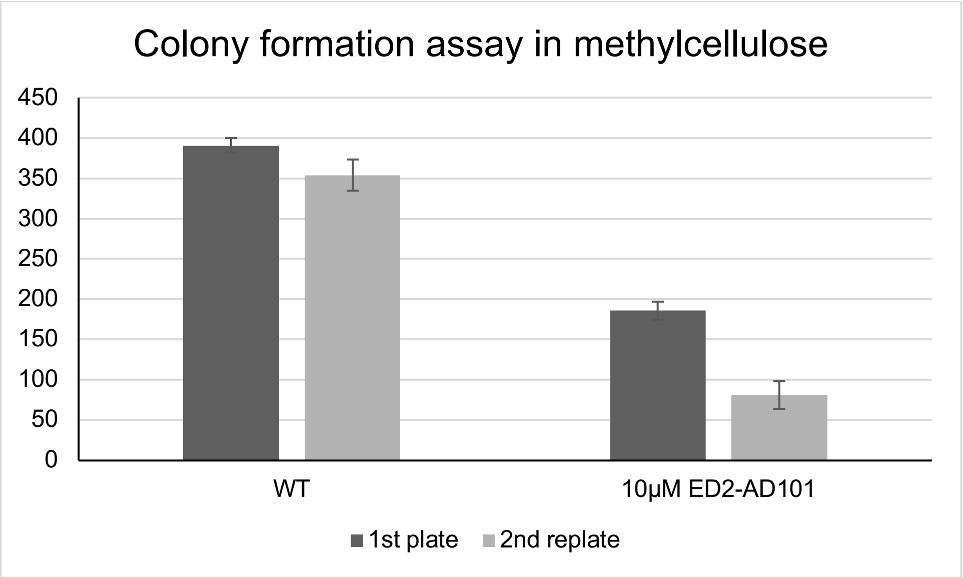
Colony formation assay in methylcellulose
450
400
350
300
250
200
150
100
50
0
WT
10µM ED2-AD101
1st plate
2nd replate

C

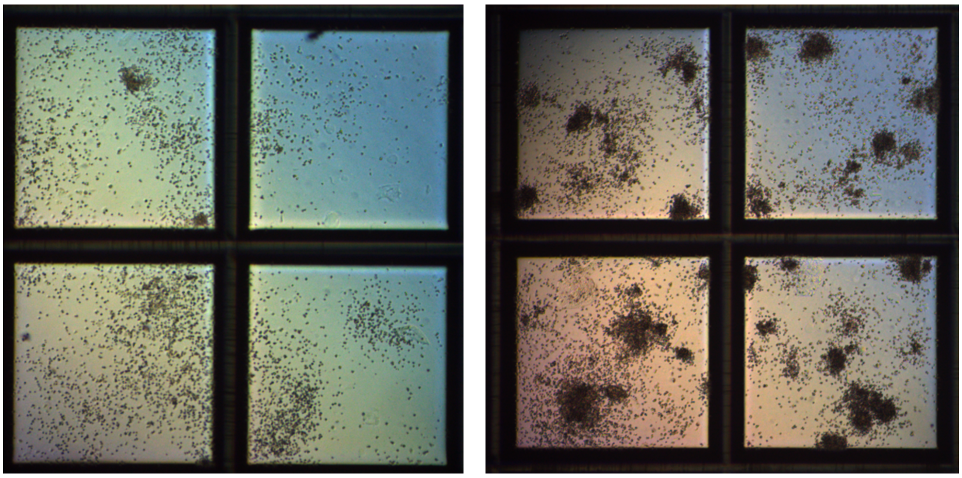

Supplementary Figure 6. The treatment of NUP98-NSD1/FLT3-ITD immortalized hematopoietic cells with ED2-AD101 inhibitor arrested their self-renewal capacity. A) IC_{50} curve calculated for the ED2-AD101 inhibitor in NUP98-NSD1/FLT3-ITD leukemic cells. B) Colony numbers after (re)plating in methylcellulose upon treatment with 10µM ED2-AD101. C) Colony formation capacity in methylcellulose after plating in presence of 10µM ED2-AD101 (left) in comparison with control (right).

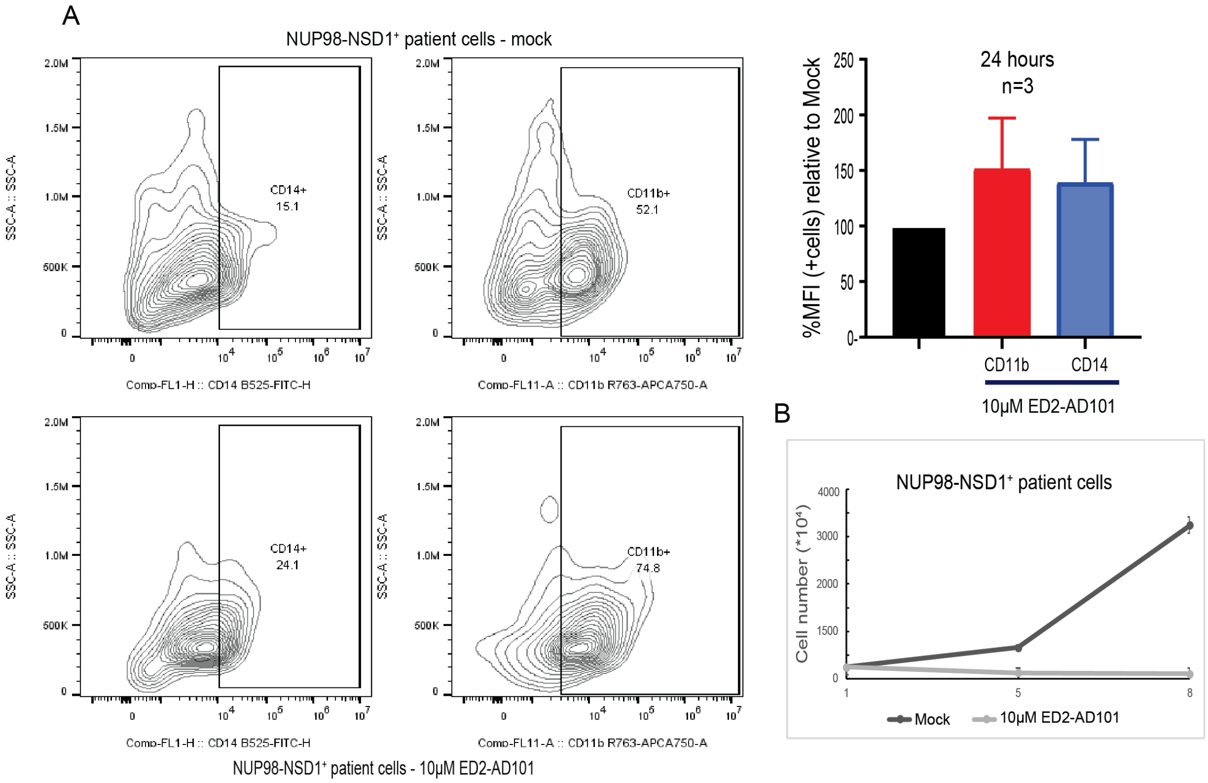

Supplementary Figure 7. Treatment of NUP98-NSD1⁺ patient cells with ED2-AD101 increased the expression of CD14 and CD11b myeloid differentiation markers. A) CD14 and CD11b protein (cell surface) expression assessed by flow cytometry in human primary patient samples with NUP98/NSD1 rearrangement after treating with SMARCA5 (10uM for 24h). Bar grafts represent the average of Mean Fluorescent Intensity of 2 independent experiments. Each bar represent standard deviation. B) Growth curve of patient cells upon treatment with 10µM ED2-AD101.

Supplementary Tables

Supplementary Table 1. List of NUP98-NSD1 interactors. Data represent volcano plot values acquired in Perseus software upon two-samples t-test (followed by Benjamini-Hochberg correction, FDR set at 0.01) comparing the log2 transformed LFQ values of the proteins bound by NUP98-NSD1 vs the control immunoprecipitation (nuclear lysate of empty Flag vector transfected cells). Significant = t-test significant; logPvalue = p value acquired upon t-test; logFC = log_2LF NUP98-NSD1/control, gene names = gene/protein names.

Significant	minus LogP	logFC	Gene name
+	2,84296286	13,3936453	NSD1
+	3,31594929	9,96846867	NUP98
+	2,01276337	9,66281509	RAE1
+	3,66105993	8,70679855	GTF3C1
+	2,69462169	8,03183746	MDN1
+	2,98679658	7,89443874	SMARCA5
+	2,92543417	7,43049049	PELP1
+	3,77865533	7,39714623	RBM14
+	1,76193486	7,30883217	ZNF198
+	2,00781825	7,21420956	XPO5
+	3,03368842	7,09086132	TRIM28
+	2,47147543	6,93023491	WDR18
+	2,01623736	6,80167961	ADNP
+	2,43572799	6,57393742	GTF3C3
+	2,87174148	6,54498196	PTPN13
+	2,11804826	6,37838745	NUP188
+	2,91963273	6,30300999	NUP93
+	2,85140258	6,13726044	BPTF
+	2,79620364	6,12250614	TEX10
+	4,55223298	5,84840488	KDM1A
+	2,37303373	5,70459461	MATR3
+	1,73935906	5,63890362	RFX1
+	1,9760425	5,61477566	CHD4
+	2,28989639	5,42981815	GTF3C2
+	2,68604772	5,28174019	RUVBL2
+	1,32674457	5,23167515	CHERP

+	1,28199147	5,21614838	ZMYM4
+	2,67529741	5,20651817	RCC2
+	2,62110797	5,16769791	QSER1
+	2,35686683	5,14248371	ATAD3A
+	3,26115275	5,09359169	HIST1H2BC
+	2,8637583	5,04381466	ELAVL1
+	2,58865868	5,00623226	RANBP5
+	2,50707942	4,99904823	RNF2
+	2,66859828	4,93769455	RBFOX2
+	2,58597503	4,84239769	NUMA1
+	2,05193479	4,69767475	EMD
+	2,447509	4,61646843	MGA
+	2,50832474	4,58588028	MOB2
+	3,32621153	4,58567143	CAD
+	2,4713552	4,56813335	SON
+	2,45645032	4,49961281	SATB2
+	3,77902208	4,48337746	GTF3C5
+	2,68637174	4,47297287	TARDBP
+	2,75489842	4,47229004	OGT
+	2,03088476	4,32345295	SMU1
+	1,28500649	4,27439594	DNAJA1
+	1,87735262	4,22990418	CBX3
+	2,47342692	4,21026421	MYBBP1A
+	1,29178159	4,13918686	CNOT1
+	1,6167023	4,13389778	RBBP7
+	2,2675333	4,12283135	ZNF326
+	3,03946314	4,11867333	U2SURP
+	2,1642145	4,11779308	GTF3C4
+	2,82240027	4,09622574	HDAC2
+	2,37279464	4,01559448	DNAJA2
+	1,4955327	3,9305048	ZFR
+	2,62068754	3,8792553	RBM17
+	2,55038825	3,86294746	SMARCC1
+	2,7828346	3,83118057	CDC73
+	1,5583045	3,81009102	PNN
+	1,97416567	3,78216076	MTA1
+	1,38600774	3,76732445	WIZ
+	0,87874341	3,75448704	PRPF40A
+	2,36657957	3,7037611	KHDRBS1
+	2,04997789	3,69710922	PRMT1
+	1,95714877	3,64979458	PHF12
+	2,23156366	3,62576103	EWSR1

+	1,58212847	3,59569836	HCFC1
+	2,00927792	3,56856918	RIF1
+	1,62030603	3,56633282	SMARCE1
+	2,92445102	3,55296707	BAG2
+	1,38387638	3,55033493	HIP1
+	2,38977278	3,44364357	MPHOSPH8
+	3,23975175	3,42827797	HDAC1
+	2,63602491	3,42826462	SF3B6
+	1,92891188	3,42787933	AP2M1
+	2,93116745	3,41767597	TCOF1
+	2,85162717	3,4090786	RBBP4
+	2,28823222	3,40544319	ATAD2
+	2,22175996	3,33126164	MCM4
+	2,32048186	3,24030018	RBM8
+	2,3444824	3,20869637	SMARCA4
+	1,94546906	3,19490528	CTBP2
+	1,36122026	3,16536903	PIK3C2A
+	0,98406484	3,16070366	SRSF9
+	2,09812893	3,14951325	PHF5A
+	2,09895084	3,14470196	PSPC1
+	1,04717116	3,10144138	TP53
+	1,50662578	3,09806061	RANBP2
+	0,93459901	3,08567047	NOP56
+	1,67759192	3,08545017	FANCI
+	2,34806894	3,07999134	SENP3
+	2,1202783	3,07937336	KHSRP
+	1,71851065	3,07212925	SNRPE
+	1,73593579	3,05214214	NCKAP1
+	0,886224	3,016469	SNRNP200
+	2,13733461	3,01312447	ZFX
+	2,18706154	3,00068665	NUP155
+	2,03305962	2,99188519	PPP1CC
+	2,11891853	2,98711491	MTA2
+	1,26693166	2,96867561	YTHDF2
+	0,67378543	2,90818977	SNRPA
+	1,47761765	2,90695286	HIST1H1E
+	1,75025206	2,80119896	UBAP2L
+	1,63652915	2,79494286	DCAF7
+	2,12815279	2,79032421	PUM1
+	2,76452112	2,7691536	U2AF2
+	1,54387364	2,76278973	PRPF19
+	0,68694481	2,74563885	KCTD5

+	1,12813665	2,73244667	MCM6
+	2,13729137	2,72563362	BUB3
+	2,23603831	2,7193327	GATAD2B
+	2,40097522	2,70379162	RALY
+	1,295204	2,69900703	IPO7
+	2,10065865	2,69591236	PRPF6
+	1,41234975	2,59932709	BAF53A
+	0,84505692	2,59724236	SRSF10
+	1,83746056	2,58689594	THOC2
+	1,16868521	2,57295132	ZNF281
+	2,82380969	2,56471348	OTUD4
+	2,05970106	2,55092716	MCM3
+	0,88145278	2,54873848	AZGP1
+	1,37708322	2,54251862	PPP2R1A
+	1,83342094	2,46431065	XPO1
+	2,06814307	2,45538902	CHD1
+	1,11356557	2,44830227	RBM25
+	1,10246848	2,35215569	USP7
+	1,67386995	2,34152699	ZNF639
+	0,59806623	2,31046486	HIST2H3PS2
+	2,26208013	2,2801342	HIST1H4H
+	0,9062736	2,26514244	BMI1
+	1,00192563	2,24540997	CPSF1
+	0,78062703	2,19855595	PPP1CA
+	2,03515969	2,19786739	RFC3
+	0,73244563	2,16501617	SMC1A
+	2,9340856	2,13310337	SAFB2
+	0,59372809	2,10571194	SRSF1
+	0,82138196	2,06678963	NME1-NME2
+	1,04662605	2,04519558	FXR1
+	3,16434201	2,01771832	PFKFB3
+	2,46326585	1,98838425	SRSF6
+	2,76078448	1,9671917	RFC4
+	1,72575416	1,96504116	NUP214
+	2,01621884	1,94750881	SPIN
+	1,17311467	1,94444275	RFC1
+	0,72183649	1,93885422	NOL9
+	1,52423874	1,93025208	TOP2A
+	1,86193124	1,89549732	NOLC1
+	0,91085285	1,83939362	THOC3
+	1,87576728	1,8264761	HDAC6
+	1,09398203	1,76181316	ZCCHC8

+	2,15440161	1,73669529	MAU2
+	3,45120355	1,70568752	RBBP6
+	2,89055769	1,68989372	CPSF3
+	1,03207883	1,66386604	JAK1
+	1,46049046	1,6589222	MCM7
+	1,25654268	1,64665794	SAP18
+	1,58725156	1,58928299	RANGAP1
+	1,55266257	1,57873631	SF3B2
+	0,39154397	1,57312679	NXF1
+	0,59639663	1,55461502	POLR2B
+	1,4221445	1,5483284	CPVL
+	1,50602086	1,5368309	NUDT21
+	0,75231583	1,5339756	MCM2
+	1,59031251	1,53395939	POLR2A
+	1,04009882	1,5257082	TARBP1
+	1,25251981	1,52348995	SF3A3
+	1,76237164	1,49924374	H3F3B
+	1,63142668	1,45345497	SUMO1
+	1,58144181	1,34489059	RBBP5
+	1,26002016	1,33974171	MCM5
+	0,60154359	1,29361534	SF3B5
+	1,02694596	1,28165722	TRIM21
+	1,84921176	1,27191925	SBSN
+	0,53303356	1,24722767	CBX1
+	1,89028045	1,22134113	PRPF31
+	0,87245151	1,17680645	KPNA6
+	1,30277168	1,16100979	NOC4L

Supplementary Table 2 List of NSD1 interactors. Data represent volcano plot values acquired in Perseus software upon two-samples t-test (followed by Benjamini-Hochberg correction, FDR set at 0.01) comparing the log2 transformed LFQ values of the proteins bound by NSD1 and control immunoprecipitation (nuclear lysate of empty Flag vector transfected cells). Significant = t-test significant; logPvalue = p value acquired upon t-test; logFC = $\log_2$ LFQ NSD1/Control gene names = gene/protein names.

Significant	minus LogP	logFC	Gene names
+	2,68301969	11,072587	NSD1

+	4,46181078	7,11112309	GTF3C3
+	2,61709665	6,95005703	GTF3C1
+	4,87122401	6,83339787	GTF3C4
+	2,19645496	6,27922154	PELP1
+	3,99500449	5,49840641	WDR18
+	4,24111821	5,32756519	GTF3C5
+	4,17800585	5,3271513	TEX10
+	1,86216543	5,23644066	MDN1
+	2,03935785	5,08235264	GNB2L1
+	1,71064225	4,82434559	GTF3C2
+	1,82791214	4,77177906	ZFX
+	2,01680997	4,44696903	NOLC1
+	4,12631753	3,82809925	NUMA1
+	2,57207281	3,69794846	MGA
+	3,10359095	3,59079456	DNAJA1
+	1,14606759	3,47417164	MYBBP1A
+	1,09660757	3,31563854	DDX18
+	3,93820913	3,10669041	UBTF
+	1,23538441	3,07138825	ATAD3A
+	2,98880903	3,02704716	TOP2A
+	3,42816304	2,95920944	NOP56
+	1,3334528	2,90404129	DDX1
+	3,5875533	2,90182018	ADAR
+	2,18954345	2,86843014	TCOF1
+	1,75896777	2,84452438	MAP1B
+	1,49252054	2,64733505	DDB1
+	2,65646612	2,63862419	MDC1
+	0,75790745	2,62948227	IGF2BP1
+	1,13223795	2,6218462	EIF3A
+	3,41684228	2,61549568	EIF3L
+	1,13464781	2,46141052	GATAD2B
+	2,44379633	2,39760304	KPNA1
+	1,31344551	2,32665634	BUB3
+	1,99164043	2,1998682	SNRPA1
+	2,19273163	2,17953873	SMARCC1
+	1,38548156	2,15889645	TCERG1
+	3,55897656	2,0709486	TOP1
+	3,74830969	2,06319332	GTF2I
+	2,21250778	2,0394907	PIP
+	2,77600427	2,03004265	POLDIP3
+	2,40471246	2,02380657	HRNR
+	1,60959092	2,0154829	SMARCA5

+	3,46975858	1,93523598	BAZ1B
+	2,58921455	1,9173727	TOP2B
+	0,87250249	1,90122509	OTUD4
+	2,58005699	1,8959856	PWP2
+	0,85210084	1,8536005	WDR3
+	1,29212152	1,77174187	PTPN13
+	1,8448937	1,75408936	HIST2H2AB
+	1,73448546	1,69106865	WDR36
+	1,27226906	1,66857338	CBX3
+	1,64552852	1,57938767	ADNP
+	3,32072447	1,52844715	EIF3C
+	0,80028882	1,52653503	ZNF326
+	1,06419282	1,48921204	SNRPF
+	1,53417214	1,48743343	SRP14
+	2,48337143	1,34657192	CTR9
+	1,02103308	1,24887466	RAD50
+	2,45051326	1,16370964	HIST1H4H
+	2,15304121	1,15651512	KIAA0100
+	1,64941455	1,15243626	HIST1H2BJ
+	1,1203601	1,12954807	HIST2H3PS2
+	1,88370742	1,11652565	HIST1H2AH
+	2,14424203	1,10961437	H2AFV
+	1,72861298	1,07586765	H2BFS
+	1,50774487	1,0566721	MCM6
+	1,92995046	0,9474678	H3F3B
+	0,84607318	0,85665512	PPIH
+	1,06502391	0,84342194	CDC73
+	0,89095518	0,82750702	RUVBL2
+	0,8622884	0,79950714	SMC1A
+	1,38542346	0,78582192	SRSF10
+	1,45652451	0,7808609	HIST1H2AB
+	0,8887756	0,75358772	YWHAG
+	1,07590215	0,73135281	ENO1
+	1,06546535	0,72688294	H2AFY
+	1,31938925	0,72018147	EEF1A1
+	0,96572936	0,69293785	CORO1C
+	1,33465711	0,67615795	SFRS5
+	2,28796368	0,66955662	SNRPD2
+	1,59753605	0,65484715	RBM15
+	2,75776943	0,65073872	HIST1H1C
+	1,76854098	0,6498661	EIF4B
+	2,89099177	0,63855076	RUVBL1

+	2,24078237	0,6194849	PARP1
+	1,2237132	0,56313229	HNRNPA0
+	1,13843504	0,55384827	H2AFY2

Supplementary Table 3. List of ΔNUP98 interactors. Data represent volcano plot values acquired in Perseus software upon two-samples t-test (followed by Benjamini-Hochberg correction, FDR set at 0.01) comparing the log2 transformed LFQ values of the proteins bound by ΔNUP98 and control immunoprecipitation (nuclear lysate of Empty Flag vector transfected cells). Significant = t-test significant; logPvalue = p value acquired upon t-test; logFC = log_2 LFQ ΔNUP98/control, gene names = gene/protein names.

Significant	minus LogP	logFC	Gene names
+	3,373267	11,116478	NUP98
+	3,18978226	10,5130348	RAE1
+	1,90371177	7,36108494	MATR3
+	2,71145159	6,84062481	KCTD5
+	1,77552134	6,7501154	MGA
+	1,65666725	5,42560577	CHERP
+	2,40982484	5,18467522	SPIN
+	3,60165613	5,01546288	RBM14
+	1,50575733	4,49457073	RBBP7
+	1,16179926	4,41981697	HIST1H1C
+	2,29471058	4,40950871	HDAC6
+	1,12237665	4,16233826	MYCBP
+	1,55264688	3,97468853	PRMT1
+	0,9610658	3,96452808	RIF1
+	3,71967707	3,78046131	OTUD4
+	1,26116526	3,66745186	CHD4
+	2,6322866	3,58026218	SATB2
+	1,979372	3,54566574	MTA2
+	1,89527952	3,39315796	ELAVL1
+	1,8505453	3,30839825	PHF5A
+	2,47187686	3,28622246	HIST1H2BC
+	1,71706089	3,22574616	GATAD2B
+	1,62132986	3,21904564	NUDT21
+	1,47718811	3,20242596	NOP14

+	1,02115576	3,17565155	WIZ
+	0,84831623	3,16346836	MOB2
+	2,25482986	3,13425541	RIOK1
+	1,53573909	3,04686356	HDAC1
+	0,9532236	3,02934933	TRIM21
+	0,98953762	3,00821018	SMARCC2
+	2,03808312	2,97165299	MAP1B
+	2,10686794	2,7777853	HCC5
+	0,89237655	2,56142235	KHDRBS1
+	1,31187813	2,29433823	NUMA1
+	0,84032696	2,26880264	SMARCA5
+	2,2780737	2,23550129	DDX48
+	2,06278067	2,19487286	SMCHD1
+	1,65937307	2,09959412	BAG2
+	0,85879968	1,97021103	SNRPA
+	1,98809214	1,96566105	S100A14
+	1,73079226	1,95915222	RBM5
+	2,67922822	1,92157269	U2AF2
+	0,97040202	1,86279678	MTA1
+	1,6742834	1,8580904	S100A16
+	1,01145241	1,50170136	OGT
+	1,80999739	1,41055679	SBSN
+	1,06571727	1,39362812	DEFA3
+	2,05458707	1,35268593	TMPO
+	1,86781769	1,17067528	PBRM1
+	1,78685704	1,16765213	U2SURP
+	0,95377206	1,12614155	PPP1CC
+	1,13311022	1,11325455	SNRNP200
+	2,40483605	1,0666523	ZBTB24
+	2,31550321	0,95722008	H3F3B
+	1,54487816	0,83014011	SMARCC1
+	1,8835791	0,81859779	SF3B3
+	1,40756883	0,81099987	BCLAF1
+	1,05963967	0,74625683	GM2A
+	1,48711087	0,73314858	CCAR1
+	3,36898117	0,72648239	DDX5
+	1,29428799	0,70002556	IGKV2D-29
+	1,72367721	0,68976498	HDAC2

Supplementary Table 4. List of FG1 repeat domain-dependent NUP98-NSD1 interactors. Data represent volcano plot values acquired in Perseus software upon

two-samples t-test (followed by Benjamini-Hochberg correction, FDR set at 0.01) comparing the log2 transformed LFQ values of the proteins bound by NUP98-NSD1 and ΔNUP98FG1-NSD1. Significant = t-test significant; logPvalue = p value acquired upon t-test; logFC = log_2 LFQ NUP98-NSD1/ΔNUP98FG1-NSD1, gene names = gene/protein names.

Significant	minus LogP	logFC	Gene names
+	6,29740038	7,87239838	XPO5
+	5,72174746	6,8057127	CNOT1
+	4,21473258	6,34400082	BPTF
+	8,75048463	6,29514551	NUP188
+	5,53517066	6,26208592	RANBP5
+	2,32848873	5,33372498	NUP93
+	5,99599719	5,3146205	AP2B1
+	4,34046895	5,27239561	HCFC1
+	4,28431579	5,03797007	ATAD2
+	5,05496933	4,99276972	TARDBP
+	1,6160229	4,90993357	MACF1
+	4,55698197	4,79953098	ZNF281
+	4,6515004	4,72536516	PIK3C2A
+	3,38947917	4,66935873	DHX9
+	3,23706213	4,49547005	PCBP1
+	4,48243266	4,47645855	EMSY
+	3,86414128	4,38044024	AP2A1
+	4,84696169	4,31837463	CYFIP1
+	1,24363181	4,29556751	HEL-S-69p
+	4,45606028	4,28251553	SKIV2L2
+	6,95871644	4,27900934	NCKAP1
+	5,45271757	4,25850105	MCM6
+	5,03880833	4,24445868	USP9X
+	3,76309232	4,16394186	XPO1
+	3,53268453	4,16314888	AKAP8L
+	4,70697068	4,14990854	BUB3
+	3,13957416	4,12153673	AP2M1
+	4,69012992	4,06453323	YTHDF2
+	5,75522307	4,01144838	KDM1A
+	4,17017456	3,98017645	SUGP2
+	4,10957403	3,88368416	NOP56
+	1,83351373	3,84807205	RFX1
+	3,06571484	3,72814846	POLDIP3

+	5,30857453	3,69647741	BMI1
+	2,81734453	3,62732983	ZNF639
+	3,4630063	3,615973	TNPO3
+	2,78720317	3,59600544	ANAPC1
+	6,94870614	3,55139875	FANCI
+	2,94915105	3,53468275	NOP58
+	4,81458981	3,51725435	OGT
+	4,43054668	3,50056458	IPO7
+	4,23519696	3,48444271	RBM22
+	3,72959817	3,42033243	NUMA1
+	3,54826187	3,36024094	KPNB1
+	3,11233102	3,29416227	mcdrh
+	1,84859071	3,25427532	RBM14
+	2,02151434	3,23023176	ZNF326
+	3,22703322	3,12889004	IQGAP2
+	2,53449369	3,06890249	ZNF198
+	1,74543723	3,06835032	QSER1
+	3,65042252	3,06032848	KDM5A
+	2,50345597	3,05715084	RFC4
+	4,9840572	3,05095959	DKFZP586J0619
+	3,49095988	3,03792095	MPHOSPH8
+	2,43112232	3,03441286	CDK1
+	4,41439963	3,01528597	HIC2
+	1,78098224	3,00394392	ZMYM4
+	2,10678223	2,99728251	HEL-S-39
+	1,55988278	2,99528408	HIST2H2AB
+	4,26083414	2,94345522	CPSF2
+	2,25705015	2,87824154	RQCD1
+	3,70164057	2,87499094	NUP155
+	4,74782848	2,86878014	HIP1
+	2,7049184	2,86405277	SF3B5
+	2,80491379	2,83862782	SMARCA5
+	2,79560024	2,8203969	RBM15
+	1,54136299	2,81885767	PHF12
+	2,88846939	2,77786541	THOC6
+	2,54235638	2,76478243	HSU53209
+	2,66513882	2,72486782	ADNP
+	2,52054941	2,67675448	TCERG1
+	5,59028	2,67355251	C17orf49
+	2,58631061	2,65840054	RBBP5
+	4,00875704	2,65827799	GEMIN4
+	3,50460405	2,65010738	SUMO1

+	3,26966023	2,64558077	TARBP1
+	3,99717627	2,62170315	CHERP
+	3,4132273	2,6176672	RBFOX2
+	1,90638519	2,61360169	FAM208A
+	1,24909475	2,56476402	HEL-S-15
+	3,32902929	2,52685928	CAPZB
+	3,19817719	2,51272917	SMARCB1
+	3,48714116	2,50621223	DKFZp566E044
+	1,23137597	2,48566341	THOC3
+	1,02287971	2,48242283	HIST1H1E
+	2,99008608	2,4354105	ADNP2
+	1,49238414	2,41332817	RALY
+	1,27717533	2,39724588	BRCA2
+	1,74457035	2,26955223	CBX1
+	0,97327539	2,25942564	ZBTB2
+	2,21173732	2,24206448	RBBP7
+	1,79531317	2,22426081	SMARCD2
+	1,57590721	2,20334387	RANGAP1
+	2,16491952	2,20156431	TIAL1
+	1,78713857	2,18697929	POLR2B
+	1,41022049	2,17627335	MAU2
+	2,20381118	2,15892506	SON
+	1,20533382	2,15603542	POLR2A
+	1,12121004	2,14434242	DDB1
+	1,11326321	2,14178896	AKAP8
+	1,0977262	2,12859058	ZFR
+	3,43862817	2,08123207	GCN1L1
+	3,37411317	2,06705761	ARID2
+	1,05141653	2,04759693	NME1-NME2

Supplementary Table 5. List of FG2 repeat domain-dependent NUP98-NSD1 interactors. Data represent volcano plot values acquired in Perseus software upon two-samples t-test (followed by Benjamini-Hochberg correction, FDR set at 0.01) comparing the log2 transformed LFQ values of the proteins bound by NUP98-NSD1 and ΔNUP98FG2-NSD1. Significant = t-test significant; logPvalue = p value acquired upon t-test; logFC = $\log_2$ LFQ NUP98-NSD1/ΔNUP98FG2-NSD1, gene names = gene/protein names.

Significant	minus LogP	logFC	Gene names
+	5,3394526	8,14285851	RBM14
+	5,9130332	7,41718721	XPO5
+	5,82705359	6,5565691	NUP188
+	6,38420604	6,54839993	BPTF
+	4,62584842	6,38214731	NUP93
+	5,3168203	6,15537071	QSER1
+	4,95223418	6,00559044	ZNF198
+	5,57073177	5,43994284	CNOT1
+	7,49304845	5,43415928	RFX1
+	6,06368322	5,38267851	NUMA1
+	4,42348503	5,14042807	ACIN1
+	4,57990762	5,09127379	RANBP5
+	4,63563993	5,05558157	RBFOX2
+	5,4248651	4,97717047	AP2B1
+	4,92523766	4,93637085	SRRT
+	6,23236693	4,90572453	CHERP
+	4,41675621	4,88272762	RIF1
+	3,74380186	4,66899586	SON
+	5,25719051	4,58917856	PPHLN1
+	4,74050602	4,46535826	ZMYM4
+	5,39377439	4,41066217	ATAD2
+	3,54983859	4,35424566	TARDBP
+	4,74553615	4,28652144	DNCL1
+	3,47783003	4,2846446	SMARCC1
+	2,53601388	4,23208714	PHF12
+	4,67502606	4,14637232	OGT
+	4,16020257	4,12588167	XPO1
+	2,79934555	4,09370709	MGA
+	6,59804755	4,05192137	SMU1
+	2,8148668	3,86201096	PDE6H
+	3,91739762	3,83349562	TP53
+	7,08722617	3,8129015	SMARCA5
+	4,20940397	3,80473566	ZNF326
+	4,0734725	3,80006599	FANCI
+	2,92324354	3,79454708	AKAP8
+	2,77523462	3,78223276	TCOF1
+	5,946748	3,77552366	ZNF281
+	4,97515061	3,77290487	HCFC1
+	3,39797297	3,73235416	SMC1A
+	5,38076454	3,7234354	IPO7

+	3,81822705	3,70199776	CYFIP1
+	4,63155527	3,66044045	SRSF10
+	4,99401559	3,62420416	U2SURP
+	1,9442642	3,5971036	ATAD3A
+	4,52260067	3,58920574	RBM8
+	3,95087239	3,53330231	YTHDF2
+	2,8757705	3,53163195	RBM25
+	5,45203117	3,47916699	MYBBP1A
+	5,03285327	3,40343046	BUB3
+	3,78811276	3,36506033	RANBP2
+	2,87528266	3,35449266	DCAF7
+	4,89256068	3,32694435	GATAD2B
+	5,03561211	3,31673288	MAP1B
+	2,79975712	3,30206633	RBM22
+	2,81978798	3,29917049	EMSY
+	1,32538318	3,27879667	H2AFV
+	1,0164449	3,25987577	MACF1
+	4,63291066	3,24360132	BMI1
+	2,37873392	3,23258686	RBBP7
+	2,93179764	3,22540855	RFC3
+	4,50893413	3,18320894	SUGP2
+	4,09315904	3,17625809	AP2M1
+	5,15295724	3,17623949	NCKAP1
+	4,87048735	3,15465927	AP2A1
+	3,7567777	3,1447525	MCM4
+	2,18986849	3,0760808	OTUD4
+	3,62085073	3,0479455	TMPO
+	2,3664858	3,02300167	ZFR
+	5,20196321	3,02248192	CHD4
+	1,64514891	3,01813412	HIST1H1C
+	1,2662883	3,01384449	CBX3
+	3,59740825	3,00109053	NOP56
+	2,81998614	2,98454142	TOP2A
+	1,64553487	2,94293022	ANKFY1
+	2,90633844	2,92177868	NOC4L
+	3,10266108	2,9002595	USP9X
+	3,75608523	2,88235045	AKAP8L
+	2,27075795	2,87908983	PRPF40A
+	2,75824303	2,831738	PHF5A
+	5,22998217	2,8118701	KCTD5
+	4,79252993	2,77990913	KDM1A
+	2,95665602	2,75497103	ZC3H18

+	4,63686969	2,69557715	GEMIN4
+	3,7108958	2,65648031	MTA1
+	3,98744464	2,64469147	TNPO3
+	3,33535468	2,60186863	IQGAP2
+	6,0947443	2,54922628	ADNP
+	1,16083791	2,54748297	NME1-NME2
+	1,04618484	2,54007387	HIST1H2AB
+	2,17528535	2,47006035	ZNF639
+	2,93177447	2,42645693	RFC1
+	1,14575548	2,42054415	HIST2H2AB
+	3,19826048	2,41634226	HIC2
+	2,43315989	2,40408945	HSU53209
+	1,04018844	2,38434744	BAG2
+	2,35372271	2,37804174	KDM5A
+	2,92273463	2,36553717	PHC3
+	2,61166271	2,35985565	POLR2B
+	2,66975974	2,32109499	NOP58
+	2,26489502	2,31598616	POLR2A
+	2,21328962	2,30093336	ANAPC1
+	2,96140946	2,29151154	HDAC2
+	1,4368497	2,28047085	SF3A3
+	3,56966028	2,27949238	SATB2
+	1,40330663	2,2766161	THOC3
+	3,24927054	2,23509264	WTAP
+	2,15211293	2,18208551	PIK3C2A
+	3,80406798	2,18042564	SKIV2L2
+	3,37033708	2,17434073	CDK1
+	3,53256386	2,13410759	PLRG1
+	3,91508375	2,12974739	MDN1
+	2,45529456	2,08636522	RNF2
+	0,87160357	2,08128405	POLDIP3
+	2,25353601	2,07457924	CLASP2
+	0,81874511	2,05679894	HIST1H1E
+	1,60142349	2,03746748	TCERG1
+	4,75006264	2,025455	TARBP1

Supplementary Table 6. List of FG(1+2) repeat domain-dependent NUP98-NSD1 interactors. Data represent volcano plot values acquired in Perseus software upon two-samples t-test (followed by Benjamini-Hochberg correction, FDR set at 0.01)

comparing the log2 transformed LFQ values of the proteins bound by NUP98-NSD1 and ΔNUP98FG(1+2)-NSD1. Significant = t-test significant; logPvalue = p value acquired upon t-test; logFC = $\log_2$ LFQ NUP98-NSD1/ΔNUP98FG(1+2)-NSD1, gene names = gene/protein names.

Significant	minus LogP	logFC	Gene names
+	3,96002757	8,36111355	RBM14
+	7,73699237	7,65695667	BPTF
+	6,14747199	7,52375078	XPO5
+	7,30072926	7,4834404	NUP188
+	5,33173225	7,42152691	NUP93
+	4,5160848	7,1605053	MDN1
+	5,59987475	6,57115841	EMSY
+	6,01104331	6,4522686	QSER1
+	5,12501917	6,43571043	CNOT1
+	4,45030049	6,23372746	NUMA1
+	6,03184868	6,23123074	RFX1
+	6,67844673	6,21988106	KDM1A
+	4,37128819	6,19475174	RANBP5
+	4,86136491	5,66422701	SON
+	4,70551637	5,51782799	ATAD2
+	2,65821016	5,40378475	ZNF198
+	3,52783199	5,32270336	PHF12
+	4,50411359	5,31767321	RBFOX2
+	4,4375373	5,21257544	RBM8
+	4,35254518	5,13132238	FAM208A
+	3,29908615	4,93683863	YTHDF2
+	4,53101402	4,91927576	AP2B1
+	4,07954176	4,90131331	XPO1
+	4,99238483	4,85330439	CYFIP1
+	5,70881674	4,80170679	ZNF281
+	4,40716607	4,70424223	AKAP8L
+	3,79165853	4,64336109	ZFR
+	3,18981788	4,57759714	AKAP8
+	2,60383097	4,52123642	EMD
+	4,76210575	4,52040243	OGT
+	4,20766532	4,41756344	SATB2
+	5,57094591	4,40934277	FANCI
+	3,75080726	4,35725641	SUGP2
+	4,41335858	4,30602408	RBM25

+	4,66794738	4,23945332	AP2M1
+	2,14138115	4,17129135	FXR1
+	3,42439261	4,100317	RANBP2
+	4,35183733	4,00348997	AP2A1
+	2,3173783	3,96490717	DNAJA1
+	3,27809505	3,95278359	ATAD3A
+	3,78506515	3,9332633	PIK3C2A
+	2,77436235	3,91746569	ZNF326
+	1,98057407	3,8972826	PNN
+	3,3591882	3,89388132	KPNB1
+	3,51551517	3,88107061	BMI1
+	2,75621933	3,87926388	TARDBP
+	4,60329902	3,84024	ZC3H18
+	4,80876609	3,78990507	NEFM
+	4,38586967	3,74150944	CDK1
+	4,01499442	3,71468306	TP53
+	4,21644877	3,70424843	USP9X
+	2,783503	3,68588352	ZNF639
+	1,64498036	3,67137003	BAG2
+	5,11837886	3,64668751	IPO7
+	3,59886905	3,62750769	NOP56
+	3,47018376	3,59895563	ANAPC1
+	3,45680917	3,59615183	TNPO3
+	6,34912534	3,54740715	NOP58
+	4,12198766	3,53708315	TARBP1
+	2,92138123	3,52603531	DDX41
+	3,84078011	3,48443985	SKIV2L2
+	3,2829659	3,4329505	POLR2A
+	3,7662771	3,38807821	PRPF40A
+	5,27327535	3,36847591	NCKAP1
+	3,21915729	3,25641871	RBM15
+	2,76760412	3,22805929	TCERG1
+	4,31652602	3,21370459	RBM22
+	3,49792426	3,17096853	MPHOSPH8
+	3,60817504	3,15410471	U2SURP
+	2,59007941	3,14882708	RFC3
+	4,85149088	3,14874029	DKFZp686A11192
+	1,2606361	3,14659786	DNAJA2
+	3,51151717	3,07855272	DCAF7
+	3,02114937	3,07517195	SMARCA5
+	2,96860371	3,05820942	KDM5A
+	2,78773408	2,98888207	C17orf49

+	3,70672247	2,986238	IQGAP2
+	2,58064787	2,92900324	ZMYM4
+	4,05016974	2,89038181	GEMIN4
+	3,54508889	2,85106802	SMARCB1
+	3,47138511	2,81318903	CDC23
+	3,83891343	2,78564072	SUMO1
+	2,62655196	2,75310993	HSU53209
+	5,72708246	2,75196505	DKFZP586J0619
+	1,19279724	2,71481466	JAK1
+	1,58804452	2,6744175	RNF2
+	3,23420774	2,57352305	MYBBP1A
+	2,27921834	2,54314899	SF3B5
+	5,1345953	2,52286911	PHC3
+	1,67488091	2,51754761	CLASP2
+	5,14611417	2,46870136	CPSF3
+	2,41532282	2,42755795	AP2S1
+	6,16143559	2,41880941	CHERP
+	2,78846443	2,4184866	HIP1
+	2,98664139	2,41272354	ACIN1
+	1,32084567	2,40450239	THOC3
+	2,29091251	2,3805995	POLR2B
+	6,08490237	2,31142235	THOC6
+	3,19244542	2,26554966	SETD2
+	3,09746385	2,23570442	GCN1L1
+	2,03596816	2,23389339	ZCCHC8
+	1,48826126	2,21806669	NUP155
+	1,8585189	2,21755266	TEX10
+	1,41213501	2,21525574	MAP1B
+	2,6885773	2,20744705	CAPZB
+	2,1758783	2,18035793	SSRP1
+	1,41146401	2,15455723	RQCD1
+	3,32218952	2,15151215	CPSF2
+	1,96259884	2,12427616	TRRAP
+	5,31830072	2,08211231	DKFZp566E044
+	2,56438008	1,95453501	RBBP5
+	2,57509077	1,9540205	CDC73
+	3,41269822	1,9196229	MCM5
+	1,66337567	1,90964794	MAU2
+	1,74313848	1,79682255	KIAA0863
+	2,16520216	1,79602623	SMARCA1
+	2,83649861	1,78323841	SPEN
+	1,52165406	1,7390995	EHMT2

+	1,51712959	1,72486019	WTAP
+	3,53422055	1,66251755	PPHLN1
+	1,77266447	1,65078354	RCC1
+	2,20711998	1,64758158	IPO4
+	2,26756172	1,58994675	FAM208B
